# Species complexes and the importance of Data Deficient classification in Red List assessments: The case of *Hylobatrachus* frogs

**DOI:** 10.1371/journal.pone.0219437

**Published:** 2019-08-14

**Authors:** Mark D. Scherz, Frank Glaw, Carl R. Hutter, Molly C. Bletz, Andolalao Rakotoarison, Jörn Köhler, Miguel Vences

**Affiliations:** 1 Zoologische Staatssammlung München (ZSM-SNSB), München, Germany; 2 Zoological Institute, Braunschweig University of Technology, Braunschweig, Germany; 3 Biodiversity Institute and Department of Ecology and Evolutionary Biology, University of Kansas, Lawrence, KS, United States of America; 4 Department of Biology, University of Massachusetts Boston, Boston, MA, United States of America; 5 Zoologie et Biodiversité Animale, Université d'Antananarivo, Antananarivo, Madagascar; 6 Hessisches Landesmuseum Darmstadt, Darmstadt, Germany; Universitat Trier, GERMANY

## Abstract

Taxonomy is the cornerstone of extinction risk assessments. Currently, the IUCN Red List treats species complexes either under a single overarching species name—resulting in an unhelpfully broad circumscription and underestimated threat assessment that does not apply to any one species lineage—or omits them altogether—resulting in the omission of species that should be assessed. We argue that taxonomic uncertainty alone, as in species complexes, should be grounds for assessment as Data Deficient (DD). Yet, use of the DD category is currently discouraged, resulting in assessments based on poor data quality and dismissal of the importance of taxonomic confidence in conservation. This policy may be leading to volatile and unwarranted assessments of hundreds of species across the world, and needs to be revised. To illustrate this point, we here present a partial taxonomic revision of torrent frogs from eastern Madagascar in the *Mantidactylus* subgenus *Hylobatrachus*. Two named species, *Mantidactylus (Hylobatrachus) lugubris* and *M*. *(H*.*) cowanii*, and several undescribed candidate species are recognised, but the application of the available names has been somewhat ambiguous. In a recent re-assessment of its conservation status, *M*. *(H*.*) lugubris* was assessed including all complex members except *M*. *(H*.*) cowanii* within its distribution, giving it a status of Least Concern and distribution over most of eastern Madagascar. After describing two of the unnamed lineages as *Mantidactylus (Hylobatrachus) atsimo* sp. nov. (from southeastern Madagascar) and *Mantidactylus (Hylobatrachus) petakorona* sp. nov. (from the Marojejy Massif in northeastern Madagascar), we show that *Mantidactylus (Hylobatrachus) lugubris* is restricted to the central east of Madagascar, highlighting the inaccuracy of its current Red List assessment. We propose to re-assess its status under a more restrictive definition that omits well-defined candidate species, thus representing the actual species to which its assessment refers, to the best of current knowledge. We recommend that for species complexes in general, (1) nominal lineages that can be confidently restricted should be assessed under the strict definition, (2) non-nominal species-level lineages and ambiguous names should be prioritised for taxonomic research, and (3) ambiguous names should be assessed as DD to highlight the deficiency in data on their taxonomic status, which is an impediment to their conservation. This would reduce ambiguity and underestimation of threats involved in assessing species complexes, and place the appropriate emphasis on the importance of taxonomy in anchoring conservation.

## Introduction

### Species complexes and the IUCN Red List

Species complexes are entities of multiple separate species-level lineages that cannot be reliably separated based on current knowledge. Often their resolution (i.e. identifying consistent differences among, and formally describing their constituent species) is hampered because they consist of cryptic lineages, that is, species-level units that are difficult if not impossible to distinguish with traditional methods, such as external morphology. Such cryptic diversity is often discovered when DNA barcoding [1] reveals that a ‘species’ consists of multiple, deeply separated genetic lineages. Further difficulties in resolving species complexes can arise from uncertainty in the application of available names when these cannot be easily assigned to any one lineage and the type material is too old or damaged to PCR-amplify DNA from it (although new opportunities are opening up with massively parallel target capture sequencing methods, e.g. [2]), in poor condition, and/or without helpful type locality. In dealing with species complexes, it is important to distinguish between a *species*, i.e. the biological unit considered an independent evolutionary lineage under any of a number of species concepts or criteria, and the *nomen* (plural *nomina*), i.e. the name we use to refer to a species. Species complexes comprise a number of species-level lineages that may be difficult to distinguish from one another. Nomina available for a species complex are often difficult to apply to a single member of the complex with any certainty. Nomina considered to represent synonyms can add considerably to this complexity, because their synonymy may have been based on the assumption of a single species. Their identity must be re-visited when the extent of the complex becomes apparent, as they have priority over new names if they apply to a certain divergent lineage included in valid nomina.

Although species complexes are difficult to quantify, there is no disputing that they are pervasive across all domains of life. This presents a major challenge to species-driven conservation, because extinction risk assessments and conservation strategies can only be as reliable as their underlying taxonomy [3, 4]. The International Union for Conservation of Nature (IUCN) Red List of Threatened Species (hereafter IUCN Red List) is a species-driven global resource for extinction risk assessments and suggestions for their conservation. It consists of assessments of species, wherein conservation-relevant data are summarised and they are assigned a status ranging from Least Concern to Critically Endangered (and two levels of Extinct) based on a series of criteria. Species for which data are inadequate to perform an assessment are classified as Data Deficient (a status that does not give any indication of threat status), and those that are not yet evaluated are not listed.

Taxonomic uncertainty of a nomen renders any conservation assessment for that name equivalently uncertain and unreliable. The IUCN Red List Guidelines [5] however state that ‘species’ should not be categorised as Data Deficient ‘simply because of this uncertainty: they should either be regarded as good species and assessed against the Red List Criteria, or not assessed for the Red List.’ (p. 77). A further provision exists specifically for species complexes: ‘Where a species name is widely accepted as containing multiple taxa that may deserve species level recognition (a ‘species complex’) AND there is insufficient information (direct or indirect) to apply the Red List Categories and Criteria, the ‘species complex’ should be listed as Data Deficient’ (p. 77). The ‘liberal use’ of the DD category is discouraged (p. 76), and indeed in many cases even a single individual can constitute sufficient data under a criterion (especially Criterion B, which pertains to geographic distribution, the presence of threats, and the spread of risk to the species) to assess a whole species.

As a result of these guidelines, species complexes are generally not being assessed as DD, but instead being assessed as though they contain a single good species, which often results in an apparently wide distribution range (e.g. [6–8]) and a status of Least Concern. On the one hand, this strategy has the benefit of including undescribed lineages that would otherwise go unassessed until described, but on the other hand it results in an assessment that is inaccurate for the species for which it is intended; it overestimates the distribution and underestimates the threat status of the one species (nomen) to which the assessment ostensibly applies, as well as all of the unnamed members of the complex included in its assessment. It also means that every taxonomic revision that resolves part of a species complex requires the threat status of the whole complex to be reassessed.

Here we present a case study for the discussion of Red List assessment of species complexes: the *Mantidactylus* subgenus *Hylobatrachus*, a clade of taxonomically challenging rheophilous mantellid frogs from Madagascar comprising a complex of more than seven species with two available names.

### *Hylobatrachus*: An enigmatic and complex clade of frogs

The genus *Mantidactylus* of the largely Madagascar-endemic neobatrachian family Mantellidae contains 31 described species. It is divided into six subgenera, *Mantidactylus* (2 species), *Brygoomantis* (11), *Maitsomantis* (1), *Hylobatrachus* (2), *Ochthomantis* (5), and *Chonomantis* (9), which are ecologically and morphologically distinct [9]. Most are found in close association with lotic water, with some (e.g. *Brygoomantis*) preferring slow and shallow streams and sometimes also nearby lentic water bodies, and others, particularly *Hylobatrachus*, preferring fast-flowing waters with rapids. Each subgenus of *Mantidactylus* hosts numerous candidate species (sensu [10, 11]), and at present at least 56 candidate species are recognised across all subgenera [12–14]. Most of these candidates are involved in species complexes, which impedes progress towards taxonomic resolution.

The subgenus *Hylobatrachus* contains riparian frogs, closely associated with fast-flowing streams where they are mostly found on and among rocks [9, 15], and are defined by their highly derived larval morphology [16]. This clade was defined as the *Mantidactylus lugubris* group by Blommers-Schlösser [17], and assumed to contain a single taxon, *Mantidactylus lugubris* (Duméril, 1853) by Blommers-Schlösser and Blanc [18]. The only further nomen associated to this groups is *Mantidactylus cowanii* (Boulenger, 1882), which was considered a junior synonym of *M*. *lugubris* by Guibé [19] and Blommers-Schlösser and Blanc [20] but resurrected as distinct species by Glaw and Vences [9]. The two currently accepted species in the subgenus *Hylobatrachus* are *Mantidactylus cowanii* and *M*. *lugubris*. While the former has a fairly precise type locality (Ankafana in the East Betsileo region), the latter was described with the imprecise locality information ‘Madagascar’, and it has been difficult to ascribe it to any genetic lineage with certainty.

Previous studies (e.g. [12–14]) have provided evidence for the presence of unrecognised lineages in the *Hylobatrachus* clade, with six candidate species defined so far. Taxonomic progress has been hampered by the morphological similarity among species, apparent variation among specimens genetically assigned to the same lineage, and lack of bioacoustic data for most lineages. So, while a taxonomic revision of the subgenus is long overdue, it remains challenging.

After a first IUCN Red List assessment, in which *M*. *lugubris* was classified as Least Concern and *M*. *cowanii* was not yet considered [21] we recently re-assessed the IUCN Red List status of *M*. *(H*.*) cowanii* and *M*. *(H*.*) lugubris* as Near Threatened and Least Concern, respectively [22, 23]. Due to relatively confident assignment of specimens to *M*. *(H)*. *cowanii*, we considered only specimens confidently assigned to that species in its assessment [22]. The assessment of *M*. *(H*.*) lugubris*, on the other hand, was done including all of the members of the rest of the species complex, following the IUCN Red List Guidelines [5, 23].

Here, we provide new data on members of the subgenus *Hylobatrachus* and their relationships, based on newly collected material and newly generated DNA sequence data, and provide formal descriptions of two of the candidate species. We then discuss the connotations of our revision for the IUCN Red List status of the species of this subgenus, and make recommendations for best practices for dealing with species complexes in IUCN Red List assessments.

## Materials and methods

Ethics statement: Approval for this study by an Institutional Animal Care and Use Committee (IACUC) was not required by Malagasy law, but all work complied with the guidelines for field research compiled by the American Society of Ichthyologists and Herpetologists (ASIH), the Herpetologists’ League (HL), and the Society for the Study of Amphibians and Reptiles (SSAR). All field research, collecting of specimens, including in situ euthanasia of specimens, were approved by the Madagascan Ministère de l’Environnement et du Développement Durable (Direction Générale des Forêts, DGF) under the permit numbers 215/16/MEEF/SG/DGF/DSAP/SCB.Re, 238-MINENV.EF/SG/DGEF/DPB/SCBLF/RECH, 285/MEADR/DEF/SEFLFB/FF/Aut, 238-MINENVEF/SG/DGEF/DPB/SCBLF, 218-MEEF/DEF/SPN/FFE/AUT, and 282/16/MEEF/SG/DGF/DSAP/SCB.Re, and exported under the permits 107N-EA04/MG17, 094C-EA03/MG04, and 105N-EA04/MG17. Specimens were anaesthetised and subsequently euthanized following approved methods (MS222 solution; approved by the American Veterinary Medical Association) that do not require approval by an ethics committee, after consultation of the animal welfare officer of TU Braunschweig.

For molecular analysis, tissue samples were taken from thigh muscle and preserved in pure ethanol. Studied specimens are deposited at the Zoologische Staatssammlung München (ZSM), the Zoologisches Forschungsmuseum Alexander Koenig (ZFMK), the Université d’Antananarivo, Département de Biologie Animale (UADBA), and the Muséum National d’Histoire Naturelle in Paris (MNHN). Field numbers FGMV, FGZC, MV and ZCMV refer to the zoological collections of F. Glaw and M. Vences; CRH to the zoological collection of C.R. Hutter.

The following morphometric measurements were taken by MV with a digital calliper to the nearest 0.1 mm: snout–vent length (SVL); maximum head width (HW); head length from tip of snout to posterior edge of snout opening (HL); horizontal tympanum diameter (TD); horizontal eye diameter (ED); distance between anterior edge of eye and nostril (END); distance between nostril and tip of snout (NSD); distance between both nostrils (NND); forelimb length, from limb insertion to tip of longest finger (FORL); hand length, to the tip of the longest finger (HAL); hindlimb length, from the cloaca to the tip of the longest toe (HIL); foot length (FOL); foot length including tarsus (FOTL); foot length (FL), and tibia length (TIBL). Hand length/body length ratio and foot length/body ratio were also calculated. Webbing formulae are given according to Blommers-Schlösser [17].

Call recordings were made in the field using various different tape and digital recorders with external microphones. Recordings were digitized at 22.05 kHz and 32-bit resolution, and computer-analysed using the software Adobe Audition 1.5. Frequency information was obtained through Fast Fourier Transformation (FFT; width 1024 points). Spectrograms were obtained at Hanning window function with 256 bands resolution. Temporal measurements are mostly given just as ranges due to small sample sizes, but from species from which more calls were available, means and standard deviation are also given in parentheses. Terminology of call descriptions follows Köhler et al. [24].

Genomic DNA was extracted from muscle tissue samples preserved in 100% ethanol using a standard salt extraction protocol [25]. We sequenced a segment of the 16S rRNA gene using primers 16SA-L and 16SB-H [26] using protocols as in Vences et al. [27]. Furthermore, a fragment of the nuclear recombination-activating gene 1 (RAG1) was amplified with primers Rag1-Manti-F1 (CGTGACAGAGTSAAAGGAGT) and Rag1-Manti-R1 (TCAATGATCTCTGGAACGTG) from Vences et al. [28], using the following PCR protocol: 120 seconds at 94°C, followed by 35 cycles of (20 s at 94°C, 50 s at 53°C, 180 s at 72°C), and 600 s at 72°C.

PCR products were cleaned with enzymatic purification: 0.15 units of Shrimp Alkaline Phosphatase (SAP) and 1 unit of Exonuclease I (New England Biolabs) incubated for 15 min at 37°C followed by 15 min at 80°C. Purified PCR products were sequenced on an automated DNA sequencer (Applied Biosystems ABI 3130XL). Sequencing reactions (10 μl) contained 0.2 or 0.3 μl of PCR product, 0.5 μl of BigDye 3.1 (Applied Biosystems) and 0.3 μmol of primer. Sequences were checked and edited, and heterozygous positions in both nuclear genes inferred, in the software CodonCode Aligner 3.7.1 (CodonCode Corporation). All newly determined sequences were submitted to GenBank (accession numbers MK447634–MK447729).

Sequences of the 16S rRNA gene were aligned with those from previous studies in MEGA 7 [29] using the MUSCLE algorithm. We determined the best-fitting substitution model (SYM+G) by the Bayesian Information Criterion in jModelTest 2.1. [30]. We computed a phylogenetic tree in MEGA 7 under the Maximum Likelihood (ML) optimality criterion under the GTR+G model (as it is the most similar to the SYM model, which cannot be implemented in MEGA). Node support was assessed with 2000 full heuristic bootstrap replicates. Uncorrected pairwise distances (p-distances) were calculated in MEGA 7.

Haplotypes of nuclear gene sequences were inferred using the PHASE algorithm implemented in DnaSP [31] and a Maximum Likelihood tree of phased sequences was calculated in MEGA 7 [29]. Haplotype networks were then reconstructed in HapViewer (Haploviewer), written by G. B. Ewing (http://www.cibiv.at/~greg/haploviewer), which infers haplotype networks applying the methodological approach of Salzburger et al. [32].

### Nomenclatural acts

The electronic edition of this article conforms to the requirements of the amended International Code of Zoological Nomenclature (ICZN), and hence the new names contained herein are available under that Code from the electronic edition of this article. This published work and the nomenclatural acts it contains have been registered in ZooBank, the online registration system for the ICZN. The ZooBank LSIDs (Life Science Identifiers) can be resolved and the associated information viewed through any standard web browser by appending the LSID to the prefix ‘http://zoobank.org/‘. The LSID for this publication is: urn:lsid:zoobank.org:pub:FAAF3075-D231-4832-8D4C-C962ADA31ADB. The journal’s eISSN is 1932–6203. The article has been archived and is available from the following repositories: PubMed Central and LOCKSS.

## Results

### Species diversity in the subgenus *Hylobatrachus* assessed by molecular markers

**Fig 1:**
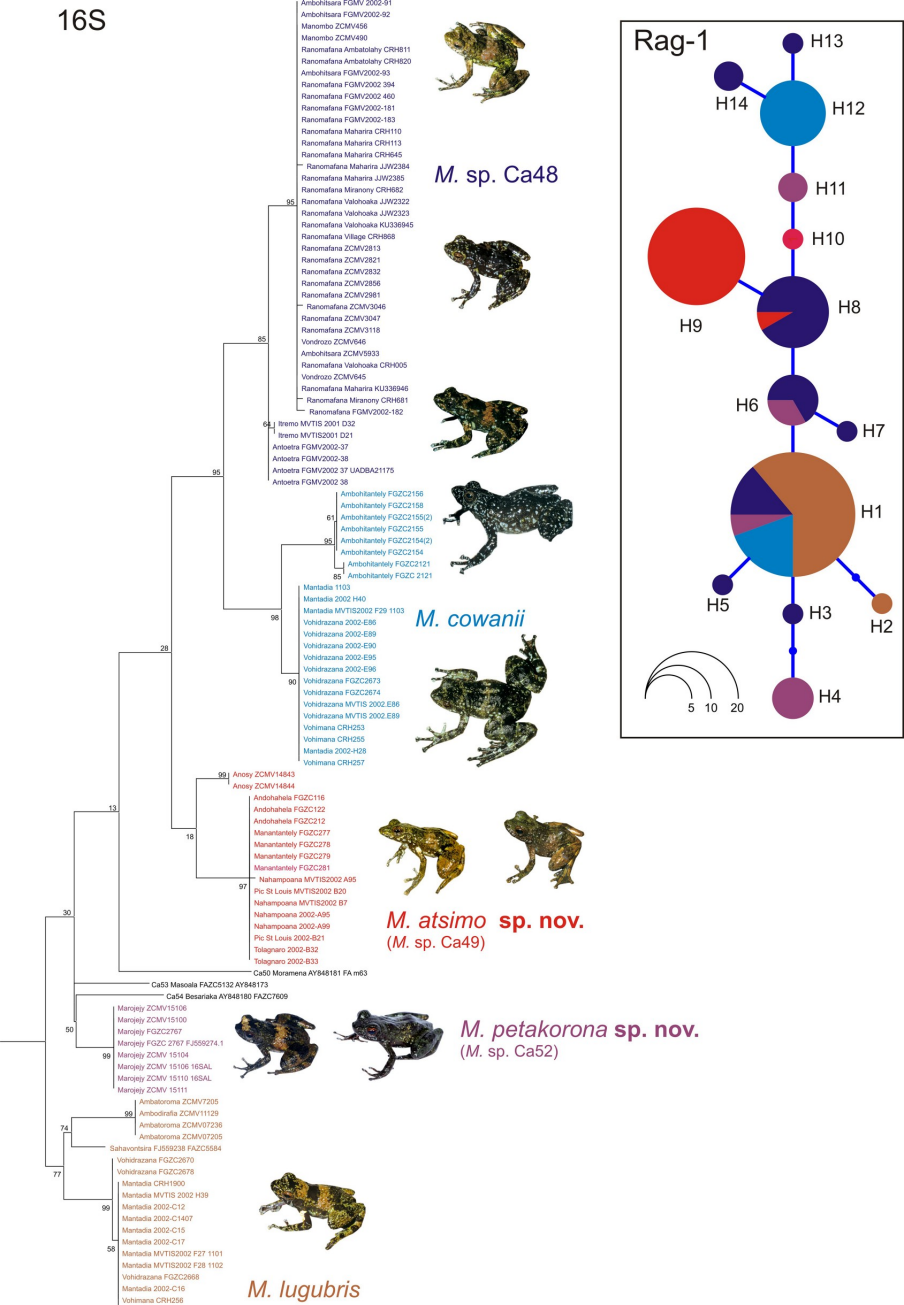
Maximum likelihood tree of 112 individuals belonging to the subgenus *Hylobatrachus*, based on DNA sequences (507 bp) of the mitochondrial 16S gene. The inset pictures show representative individuals of the respective species. Values at nodes are support values in percent of a bootstrap analysis (2000 replicates). The tree was rooted with *Mantidactylus femoralis* (subgenus *Ochthomantis*) as the outgroup (removed from the graphic for better visualization of ingroup relationships). The inset haplotype network is based on haplotypes inferred from 572 bp of the nuclear RAG1 gene for 54 individuals (haplotypes numbered H1–H14). Colours correspond to those used in the tree.

**Fig 2:**
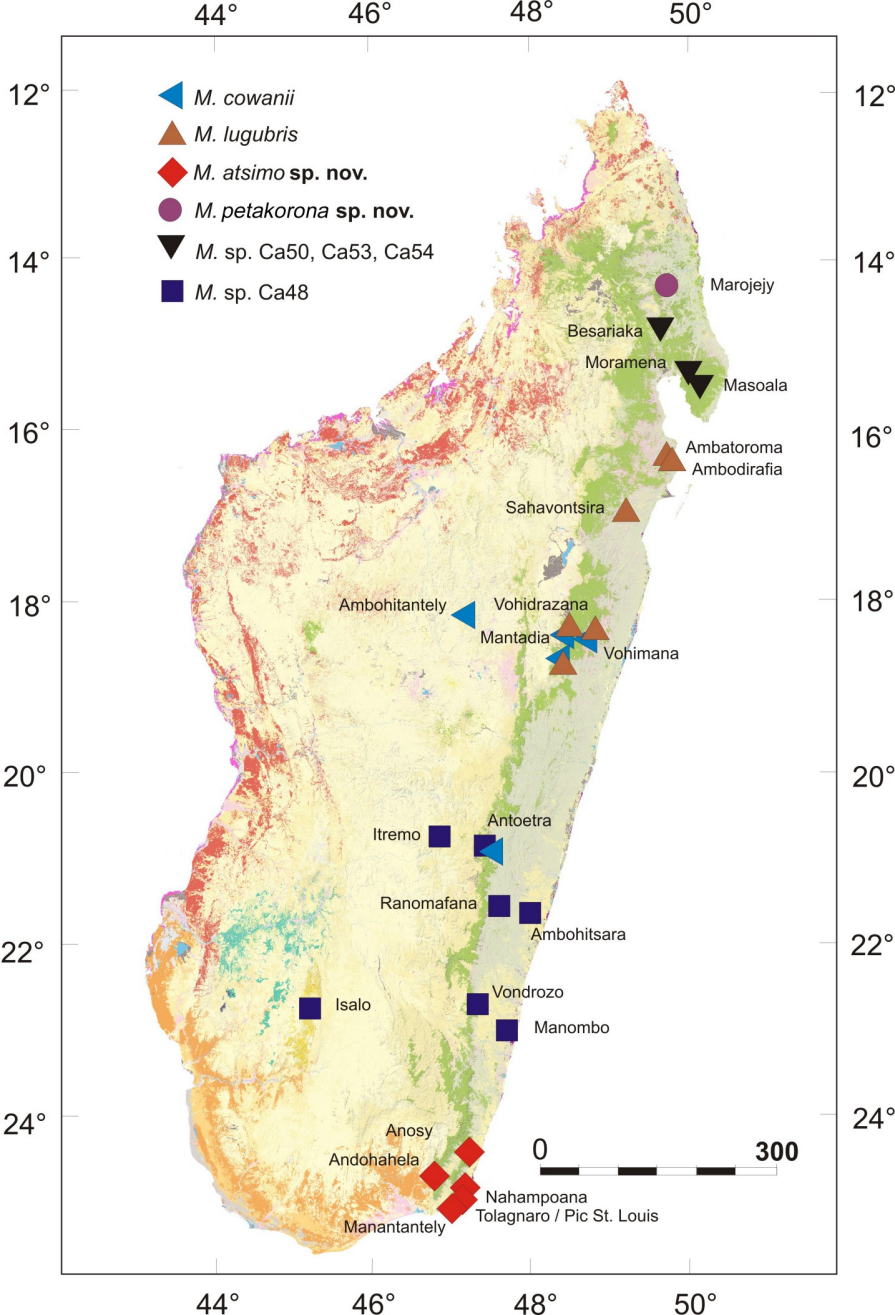
Map of Madagascar showing the known distribution of *Mantidactylus* species and candidate species in the subgenus *Hylobatrachus*. Only records confirmed by molecular data in Fig 1 are shown, except the *M*. *cowanii* record in Antoetra (see text) and the *M*. sp. Ca48 record from Isalo (molecular data in [33]). The base map is the USGS SRTM 1-Arc second digital elevation model.

Our DNA sequence alignment of the 16S rRNA mitochondrial gene consisted of 507 bp for 112 individuals of the subgenus *Hylobatrachus*. The obtained ML tree (Fig 1) confirmed the eight previously defined species and candidate species in the subgenus as deep mitochondrial lineages, several of which had additional geographic structure (Figs 1 and 2). Note that the purpose of this single-marker tree was not to resolve the deep relationships of *Hylobatrachus* but to assign specimens to distinct lineages.

**Fig 3:**
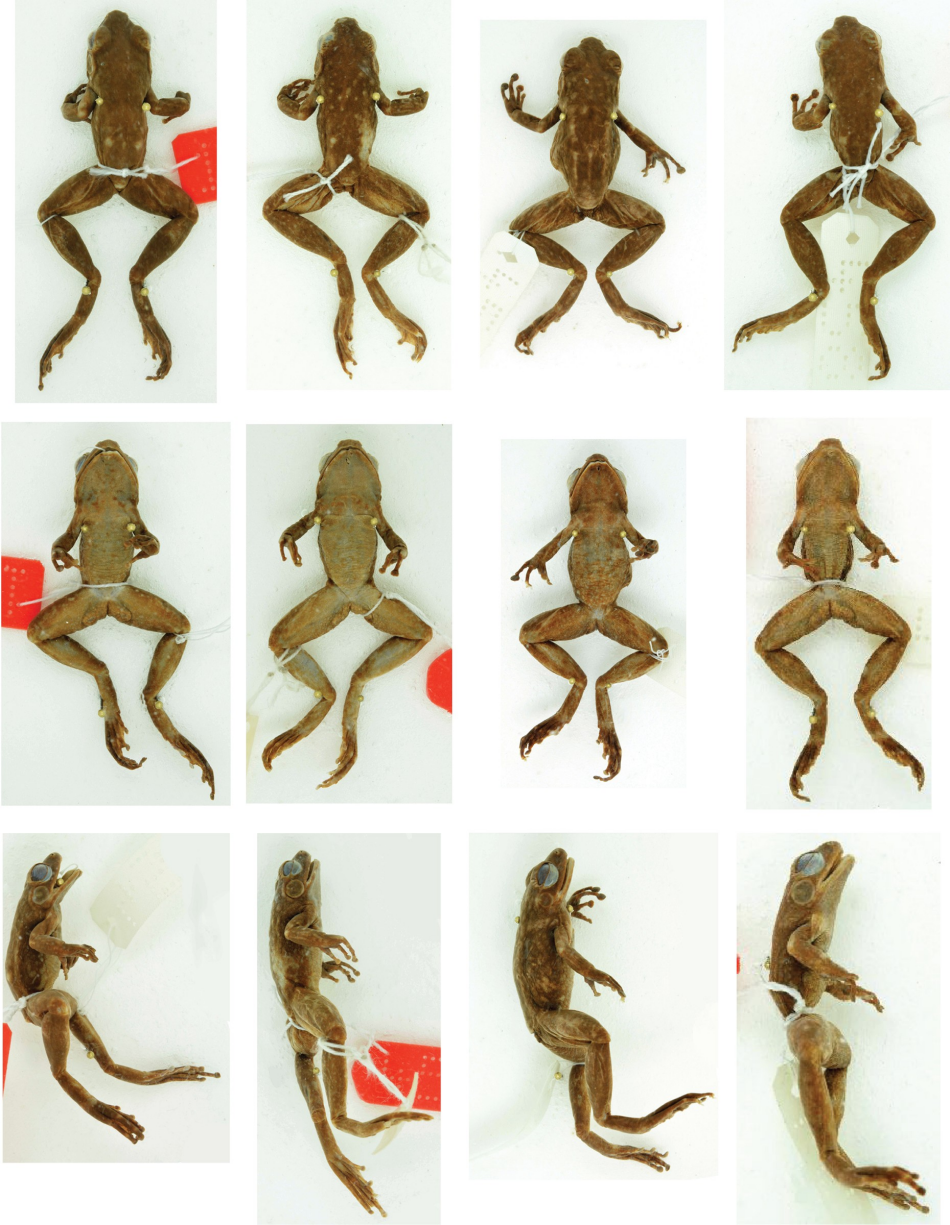
Preserved syntype specimens of *Mantidactylus lugubris* from MNHN database. MNHN 1994.1752, 4583, MNHN 1994.1750, MNHN 1994.1751 are presented (left to right) in dorsal (top), ventral (middle) and lateral (bottom view).

**Fig 4:**
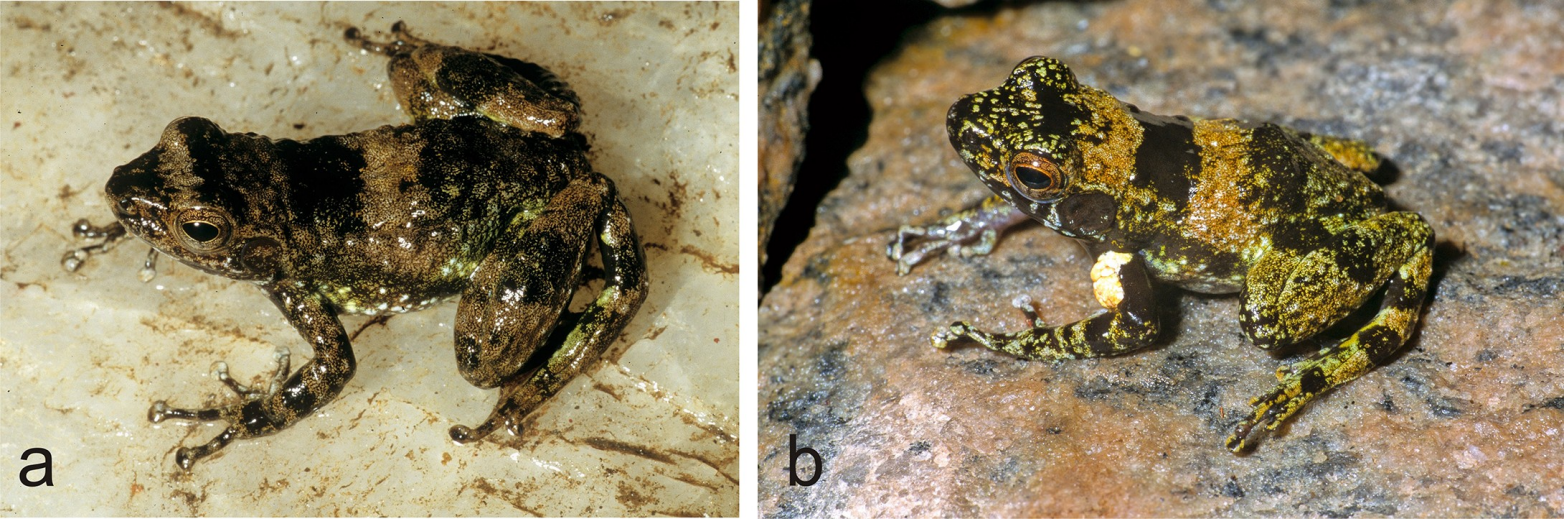
Specimens assigned to *Mantidactylus lugubris* in life. (a) Vohidrazana, (b) Mantadia.

**Fig 5:**
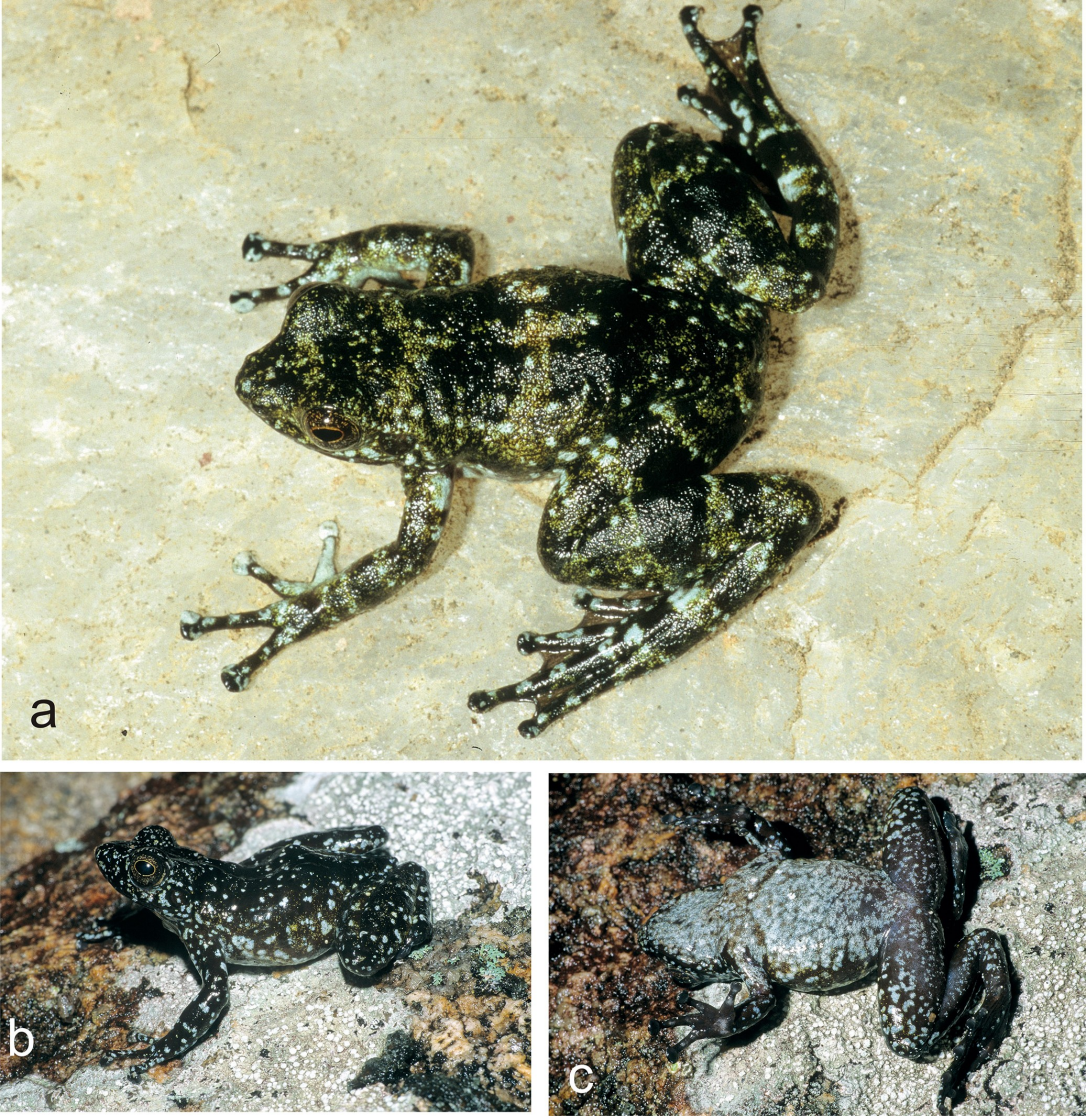
Specimens assigned to *Mantidactylus cowanii* in life. (a) Vohidrazana, (b, c) Antoetra (Soamazaka).

Three candidate species from northern Madagascar (*Mantidactylus* sp. Ca50, Ca53, and Ca54) were represented by single samples only and will not be discussed in detail in this study. Clades assigned to the two nominal species, *Mantidactylus cowanii* and *M*. *lugubris* (see section Identity of described taxa in the subgenus *Hylobatrachus* below for justification of assignment), comprised samples from multiple locations: for *M*. *cowanii*, specimens from Ambohitantely were placed in a separate subclade, sister to the subclade with samples from Mantadia, Vohidrazana, and Vohimana; for *M*. *lugubris*, specimens from northeastern coastal localities (Befanjana forest: Ambodirafia and Ambatoroma) formed one clade, a sample from another northeastern locality (Sahavontsira) formed a second clade, and specimens from the northern central east (Mantadia, Vohidrazana, and Vohimana) formed a third clade. Note that at the latter three localities, our data suggest syntopic co-occurrence of *M*. *cowanii* and *M*. *lugubris*, and this was also corroborated for these sites by morphological comparison of the voucher specimens (Table 1) which showed the differences in colour pattern and partly in body size characteristic for these species, with *M*. *cowanii* being usually characterised by being larger and having a darker dorsal colour with irregular light spotting (see Figs 3–5 and Table 1; Vohimana specimens not measured but confirmed by CRH).

**Table 1:** Morphometric data of examined specimens of *Hylobatrachus* (all in mm). For abbreviations of measurements, see Methods. Additional abbreviations: M, male; F, female, HT, holotype, ST, syntype. The column 16S indicates individuals for which a fragment of the 16S rRNA gene has been sequenced and included in the phylogenetic tree (Fig 1). Since the femoral glands in *Hylobatrachus* are usually poorly recognizable they were excluded from the measurements. The sex of the individuals was determined examination of femoral glands or by incision.

Voucher number	Field number (tissue number)	Sex	16S	Location	SVL	HW	HL	TD	ED	END	NSD	NND	FORL	HAL	HIL	FOTL	FOL	TIBL
***M*. *lugubris***																		
MNHN 1994.1750 (ST)	—	F		Madagascar	38.0	11.6	13.7	2.9	4.8	3.1	1.8	3.4	21.7	10.7	59.7	26.4	18.3	17.5
MNHN 1994.1751 (ST)	—	M		Madagascar	31.5	10.3	11.9	3.6	4.3	3.0	1.3	3.3	19.0	9.4	49.4	22.5	15.6	15.1
MNHN 1994.1752 (ST)	—	M		Madagascar	32.0	10.3	11.5	3.7	4.1	3.0	1.7	2.8	19.0	9.4	51.6	23.8	16.3	15.6
MNHN 4583 (ST)	—	M		Madagascar	32.0	11.2	11.5	3.9	4.3	3.0	1.6	3.8	21.1	9.8	54.3	25.6	17.6	16.4
ZSM 166/2002	MV 2001.1101	F	+	Mantadia	35.7	10.9	12.7	3.2	4.5	3.5	2.0	3.7	22.4	11.2	59.9	28.3	19.7	17.9
ZSM 167/2002	MV 2001.1102	M	+	Mantadia	33.6	11.3	13.0	4.1	4.5	3.3	1.8	3.1	21.4	10.6	54.8	26.4	18.1	16.8
ZSM 750/2009	ZCMV 7206	M	+	Ambatoroma	33.6	11.0	12.7	3.8	4.8	3.5	1.8	3.6	19.4	9.6	48.2	23.2	16.4	15.2
ZSM 749/2009	ZCMV 7205	M		Ambatoroma	31.3	10.3	11.8	3.7	4.6	2.9	1.9	3.6	19.3	9.5	50.4	23.1	16.1	15.2
ZSM 751/2009	ZCMV 7236	M	+	Ambatoroma	31.9	11.2	12.0	3.4	4.8	2.8	2.4	3.8	19.5	9.1	50.0	21.5	13.8	15.6
ZSM 747/2009	ZCMV 11124	F	+	Ambodirafia	35.8	13.3	13.7	3.5	5.3	3.0	2.1	3.5	22.2	10.8	58.3	26.8	18.8	17.6
ZSM 748/2009	ZCMV 11129	F	+	Ambodirafia	36.8	12.0	13.0	3.0	5.4	3.4	1.7	3.9	22.6	10.8	56.1	25.7	18.1	17.1
ZSM 65/2002	MV 2001.1364 (2002-H39)			Mantadia	36.0	13.0	14.7	3.8	4.7	3.5	2.2	3.9	NM	10.7	NM	NM	18.3	18.9
ZSM 299/2005	FGZC 2668	F		Vohidrazana	36.2	12.7	14.2	3.6	5.4	3.8	2.2	3.7	24.1	11.2	56.3	28.5	19.3	17.8
ZSM 300/2005	FGZC 2670	F		Vohidrazana	36.8	12.1	14.0	3.9	5.5	3.3	2.2	4.1	21.3	11.0	56.1	27.4	17.4	17.6
***M*. *cowanii***																		
ZSM 63/2002	MV 2001.1353 (2002-H28)	F		Mantadia	43.5	14.1	15.6	2.9	5.3	3.9	2.7	4.1	28.5	12.9	67.1	28.6	17.8	22.3
ZSM 64/2002	MV 2001.1365 (2002-H40)	M		Mantadia	32.9	11.2	12.8	3.7	4.1	3.1	1.9	2.7	21.0	8.8	54.3	24.1	12.7	16.6
ZSM 306/2005	FGZC 2121	F	+	Ambohitantely	41.3	13.6	14.8	3.3	5.2	3.8	2.1	3.9	23.2	11.8	62.4	28.8	19.5	20.2
ZSM 301/2005	FGZC 2673	M		Vohidrazana	35.7	12.2	13.5	3.3	4.9	3.6	2.3	4.3	23.1	9.8	60.8	27.7	18.1	19.5
ZSM 302/2005	FGZC 2674	M		Vohidrazana	34.2	11.9	12.2	3.9	4.2	2.9	2.2	3.6	21.1	9.2	55.3	26.2	17.1	18.2
ZSM 171/2002	MV 2001.1103 (2002-F29)	M	+	Mantadia	40.4	13.7	14.3	3.2	5.1	3.5	2.5	4.0	25.3	12.5	68.4	31.5	21.6	20.7
ZSM 297/2005	FGZC 2155	F		Ambohitantely	38.7	12.8	13.1	2.9	4.2	3.2	2.1	3.4	22.9	11.8	62.5	29.1	20.2	19.7
ZSM 298/2005	FGZC 2158	F		Ambohitantely	39.9	13.1	13.5	2.8	4.3	3.3	2.6	4.0	22.1	11.1	64.9	29.5	20.7	19.7
ZSM 296/2005	FGZC 2154	M		Ambohitantely	31.9	11.1	11.5	2.9	4.3	3.2	2.0	3.8	19.8	9.3	54.0	23.8	16.4	16.1
***M*. *petakorona* sp. nov.**																		
ZSM 305/2005	FGZC 2767	F?	+	Marojejy	29.0	10.1	10.8	2.6	3.5	2.8	1.5	3.1	19.3	8.9	53.9	25.3	14.9	17.1
ZSM 501/2016	ZCMV 15100	M		Marojejy	31.3	10.0	11.6	3.7	4.9	3.0	1.8	3.1	19.6	8.8	52.8	24.2	14.7	16.1
ZSM 502/2016	ZCMV 15104	F	+	Marojejy	34.0	10.1	11.7	2.9	4.9	2.8	1.7	3.1	20.4	8.2	58.2	26.0	17.8	16.9
ZSM 503/2016	ZCMV 15106	M	+	Marojejy	28.9	9.2	10.6	3.4	4.4	2.7	1.5	2.5	18.3	8.4	46.9	22.5	14.4	15.3
ZSM 504/2016 (HT)	ZCMV 15110	F	+	Marojejy	34.0	11.4	12.0	2.8	6.3	2.6	2.1	3.3	20.7	9.7	57.7	26.2	16.5	18.3
ZSM 505/2016	ZCMV 15111	F	+	Marojejy	27.0	9.2	10.1	2.0	4.8	1.9	1.5	2.2	17.7	8.6	50.4	23.0	13.9	16.1
***M*. *atsimo* sp. nov.**																		
ZSM 149/2004	FGZC 277	F	+	Manantantely	34.8	11.7	12.3	2.7	4.6	3.8	1.8	3.1	22.0	10.7	56.7	26.0	17.5	17.5
ZSM 150/2004	FGZC 281	F		Manantantely	33.7	11.9	12.5	2.4	4.6	3.8	2.1	3.5	20.1	10.0	50.6	24.0	16.9	15.9
ZSM 72/2004	FGZC 122	F	+	Andohahela	34.6	11.9	12.3	2.6	4.3	3.7	1.6	2.9	21.0	10.1	56.2	25.0	16.9	16.9
ZSM 69/2004 (HT)	FGZC 116	F	+	Andohahela	34.5	11.8	12.8	2.2	5.4	3.5	2.0	3.7	20.7	10.3	53.3	24.2	16.7	16.1
ZSM 174/2002	MV 2001.1483 (2002-B20 = B21)	F	+	Pic St. Louis	33.8	11.9	12.2	2.7	4.8	3.4	1.6	3.1	19.7	9.6	53.6	23.4	15.7	16.1
ZSM 253/2002	2002-A95/A99/B7	F	+	Nahampoana	31.2	10.5	11.4	2.2	4.3	3.4	1.6	3.0	18.6	8.9	52.2	23.3	15.5	16.6
ZSM 172/2002	MV 2001.1476 (2002-A95/A99/B7)	F	+	Nahampoana	34.1	11.7	12.6	2.2	4.9	3.2	1.7	2.8	21.0	9.6	56.1	24.7	16.6	16.6
ZSM 367/2016	ZCMV14843	M	+	Anosy Massif	25.2	9.9	11.4	3.0	5.2	3.0	1.8	3.0	17.4	8.7	46.3	21.2	11.0	15.0
ZSM 368/2016	ZCMV14844	M	+	Anosy Massif	28.0	10.2	12.0	3.3	5.0	3.0	2.0	2.8	17.0	8.3	47.7	21.8	14.3	14.9
***M*. sp. Ca48**																		
ZSM 493/2006	ZCMV 2821	M		Ranomafana	31.5	10.4	12.5	3.6	4.5	2.9	1.9	2.5	19.0	9.0	49.0	22.9	14.7	16.5
ZSM 494/2006	ZCMV 2832	F		Ranomafana	37.8	13.1	14.8	2.7	5.6	3.2	2.5	2.5	24.9	10.7	62.7	28.5	18.9	18.6
ZSM 495/2006	ZCMV 2856	F		Ranomafana	36.1	13.6	14.5	3.2	5.7	3.5	2.2	2.9	23.8	11.0	62.0	27.9	17.5	19.0
ZSM 496/2006	ZCMV 2981	M		Ranomafana	26.9	10.2	11.8	3.1	4.5	2.1	1.5	3.3	19.4	8.9	52.2	23.1	13.6	15.7
ZSM 497/2006	ZCMV 3046	M		Ranomafana	26.0	9.4	11.6	2.8	4.7	2.4	2.0	2.6	16.2	7.4	45.1	20.9	14.5	14.4
ZSM 498/2006	ZCMV 3047	F		Ranomafana	38.1	15.2	14.8	2.9	4.7	3.6	2.2	2.9	22.4	11.0	58.2	27.2	18.4	17.8
ZSM 499/2006	ZCMV 3118	M		Ranomafana	29.7	11.1	12.1	3.2	4.7	3.2	1.6	3.2	20.8	9.5	52.4	25.3	15.7	17.6
ZSM 717/2003	FGMV 2002.0379	?		Ranomafana	30.0	10.6	12.3	3.6	5.2	3.0	1.6	3.6	18.2	9.5	49.0	22.7	15.3	15.1
ZSM 718/2003	FGMV 2002.0385	F		Ranomafana	36.5	12.2	12.7	4.1	5.0	2.9	2.3	4.6	21.0	10.3	58.7	27.2	15.1	17.7
ZSM 719/2003	FGMV 2002.0386	M		Ranomafana	29.2	10.3	12.3	3.2	4.6	2.9	2.0	3.2	18.7	9.3	52.3	24.1	14.3	15.2
ZSM 720/2003	FGMV 2002.0391	F	+	Ambohitsara	38.0	12.5	14.5	3.1	5.8	3.0	1.8	3.1	23.2	10.9	65.7	28.9	17.9	19.5
ZSM 721/2003	FGMV 2002.0392	M	+	Ambohitsara	28.6	10.9	12.7	3.5	5.0	2.9	2.0	3.2	19.4	8.8	50.6	23.1	15.0	16.1
ZSM 722/2003	FGMV 2002.0394	M		Ranomafana	30.1	10.9	12.0	2.4	4.8	3.2	2.1	4.5	20.6	9.4	57.2	26.1	16.7	17.7
ZSM 898/2006	ZCMV 2813	F		Ranomafana	39.0	12.2	13.9	3.1	5.0	3.5	2.9	2.6	23.7	11.3	61.0	28.2	19.3	18.8
ZSM 2412/2007	ZCMV 5933	M?		Ambohitsara	26.7	9.7	11.2	3.7	3.6	3.2	1.3	2.8	18.6	8.5	47.5	21.0	10.9	14.6
ZSM 730/2003	FGMV 2002.0454	M		Ranomafana	26.8	10.3	11.8	2.8	5.0	2.9	1.8	3.2	19.3	7.9	48.7	23.2	13.2	19.5
ZSM 734/2003	FGMV 2002.0460	M		Ranomafana	27.7	9.8	11.8	3.4	3.9	2.8	1.9	4.4	18.9	8.0	51.2	23.1	14.2	15.2
ZSM 646/2003	FGMV 2002.181	F	+	Ranomafana	37.2	12.0	13.4	2.7	5.2	3.4	2.3	3.7	22.4	11.4	59.1	27.8	19.1	18.3
ZSM 744/2001	MV 2001.467 (2001-D21?)	M	+?	Itremo	26.8	8.9	9.5	2.2	3.9	2.1	1.6	2.4	16.6	8.1	44.9	20.8	13.5	13.7
ZSM 745/2001	MV 2001.468 (2001-D21?)	M	+?	Itremo	27.4	8.9	10.1	3.1	3.9	2.5	1.6	3.2	16.9	8.0	44.3	20.1	13.8	13.4

Three additional lineages in our phylogenetic tree were represented by multiple individuals: (1) one lineage from the Marojejy Massif in the northeast, corresponding to *M*. sp. Ca52 [12]); one lineage from several localities in the southeast (Andohahela, Manantantely, Nahampoana, Tolagnaro/Pic St. Louis, and probably Anosy Mountains) corresponding to *M*. sp. Ca49; (3) and one lineage from various sites in southern central Madagascar, corresponding to *M*. sp. Ca48. All of these lineages, as well as *M*. *lugubris* and *M*. *cowanii*, were supported by bootstrap support values >70%, except for *M*. sp. Ca49 where most individuals had near-identical sequences and were placed in a highly supported clade (bootstrap proportion 97%), but the placement of the two specimens from the Anosy mountains was unsupported (18%) and remains tentative.

Genetic divergences among the main lineages in *Hylobatrachus* were high. 16S uncorrected p-distance divergences as reported in Table 2 were 3.6–7.6%. The highest divergence (7.6%) corresponded to the sympatric species pair, *M*. *cowanii* and *M*. *lugubris*.

**Table 2:** Mean uncorrected pairwise distances among species and candidate species of *Hylobatrachus* in a fragment of 507 bp of the mitochondrial 16S rRNA gene.

	*M*. *lugubris*	*M*. *cowanii*	*M*. *atsimo* (Ca49)	*M*. *petakorona* (Ca52)	*M*. sp. Ca48	*M*. sp. Ca50	*M*. sp. Ca53
*M*. *cowanii*	7.6						
*M*. *atsimo* (Ca49)	4.7	6.1					
*M*. *petakorona* (Ca52)	3.9	7.1	5.5				
*M*. sp. Ca48	7.5	4.5	5.2	6.8			
*M*. sp. Ca50	6.7	8.5	5.9	5.8	6.8		
*M*. sp. Ca53	4.5	6.7	5.4	3.6	6.7	5.8	
*M*. sp. Ca54	5.0	7.1	6.0	3.4	6.3	6.0	4.2

The alignment of the nuclear gene fragment, RAG1, consisted of 572 nucleotide positions for 54 individuals of *Hylobatrachus*. The haplotype network reconstructed from these sequences contained 14 haplotypes (H1–H14 in Fig 1) which, however, did not reveal a pattern of differentiation consistent with the mitochondrial tree. Every lineage showed haplotype sharing with at least one other lineage, and one haplotype (H1) was found in four of the lineages. However, some haplotypes were more common in some species; for instance, most individuals of *M*. sp. Ca49 had one exclusive RAG1 haplotype not shared with any other of the lineages (H9), and a large proportion of *M*. *cowanii* sequences corresponded to one haplotype exclusive for that species (H12).

The extensive haplotype sharing in RAG1 might indicate incomplete lineage sorting or limited gene flow among species and candidate species of *Hylobatrachus*. This necessarily hampers species delimitation which, based on the available data, cannot rely on the genealogical concordance criterion [34]. Yet, the sympatric occurrence of *M*. *lugubris* and *M*. *cowanii* (Fig 2) and of *M*. *cowanii* and *M*. sp. Ca48 at Antoetra (Fig 2) where individuals can clearly be recognised by morphology (body size and colour pattern) clearly supports the existence of more than one species in the subgenus. While the available evidence for multiple species is weaker in *Hylobatrachus* than in other groups of recently revised Malagasy anurans (e.g. [35, 36]) we are still convinced that in light of the available evidence, a taxonomic hypothesis dividing the subgenus into various species reflects biological reality better than a one-species or two-species hypothesis—especially in light of the high divergences in mitochondrial DNA identified within *Hylobatrachus*. As a first step, we here decided to formally recognise the geographically most separated lineages, *M*. spp. Ca49 and Ca52, as distinct species, given that these also show some consistent morphological differentiation from the other lineages, as presented in detail in the diagnoses below.

### Identity of described taxa in the subgenus *Hylobatrachus*

**Fig 6:**
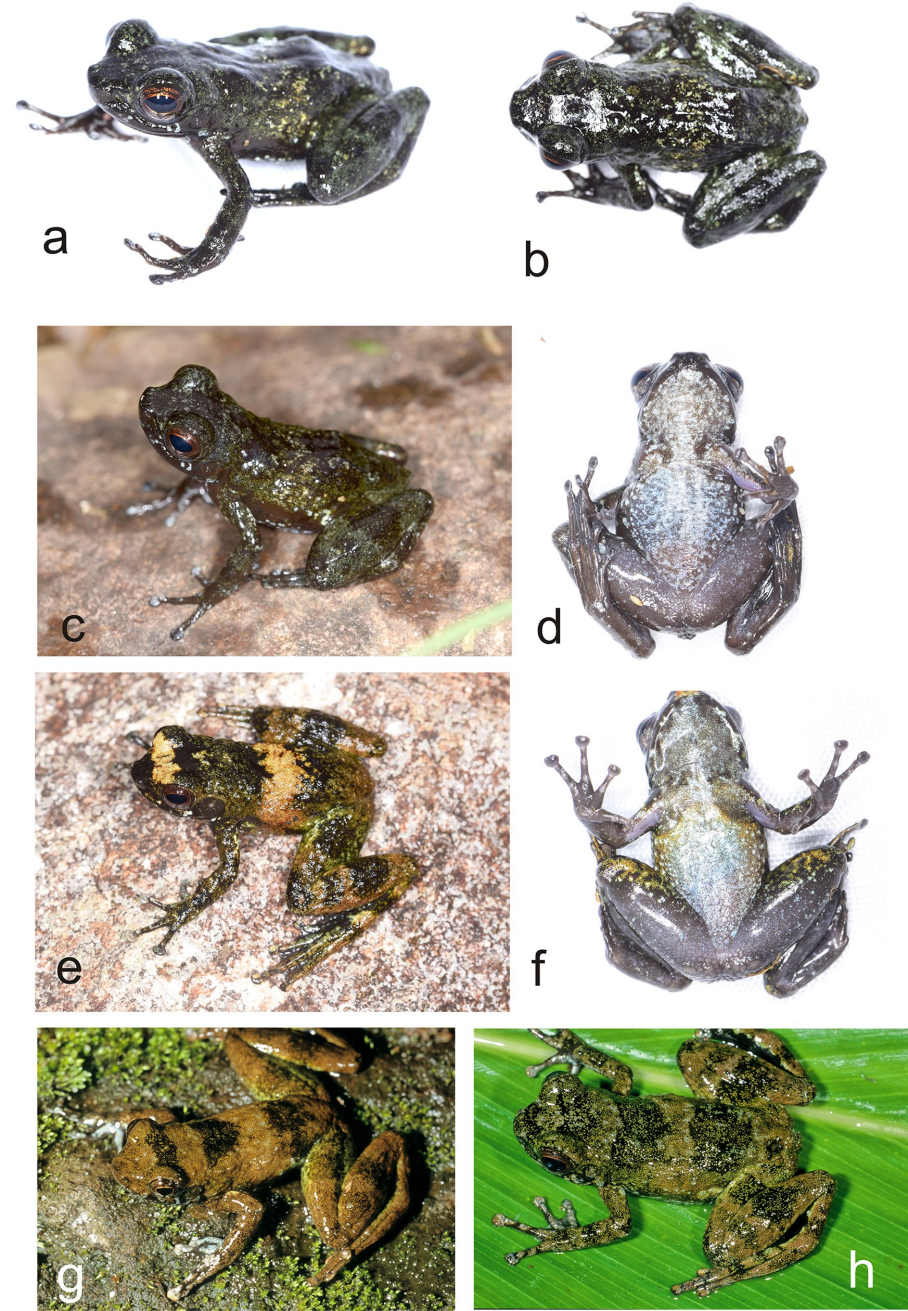
Specimens of *Mantidactylus petakorona* sp. nov. from Marojejy (low elevation localities around Camp ‘Mantella’) in life. (a-d) Holotype ZSM 504/2016, (e-f) paratype ZCMV 15121, (g) paratype ZSM 305/2005, (h) probably paratype ZFMK 59909, photographed in 1995.

**Fig 7:**
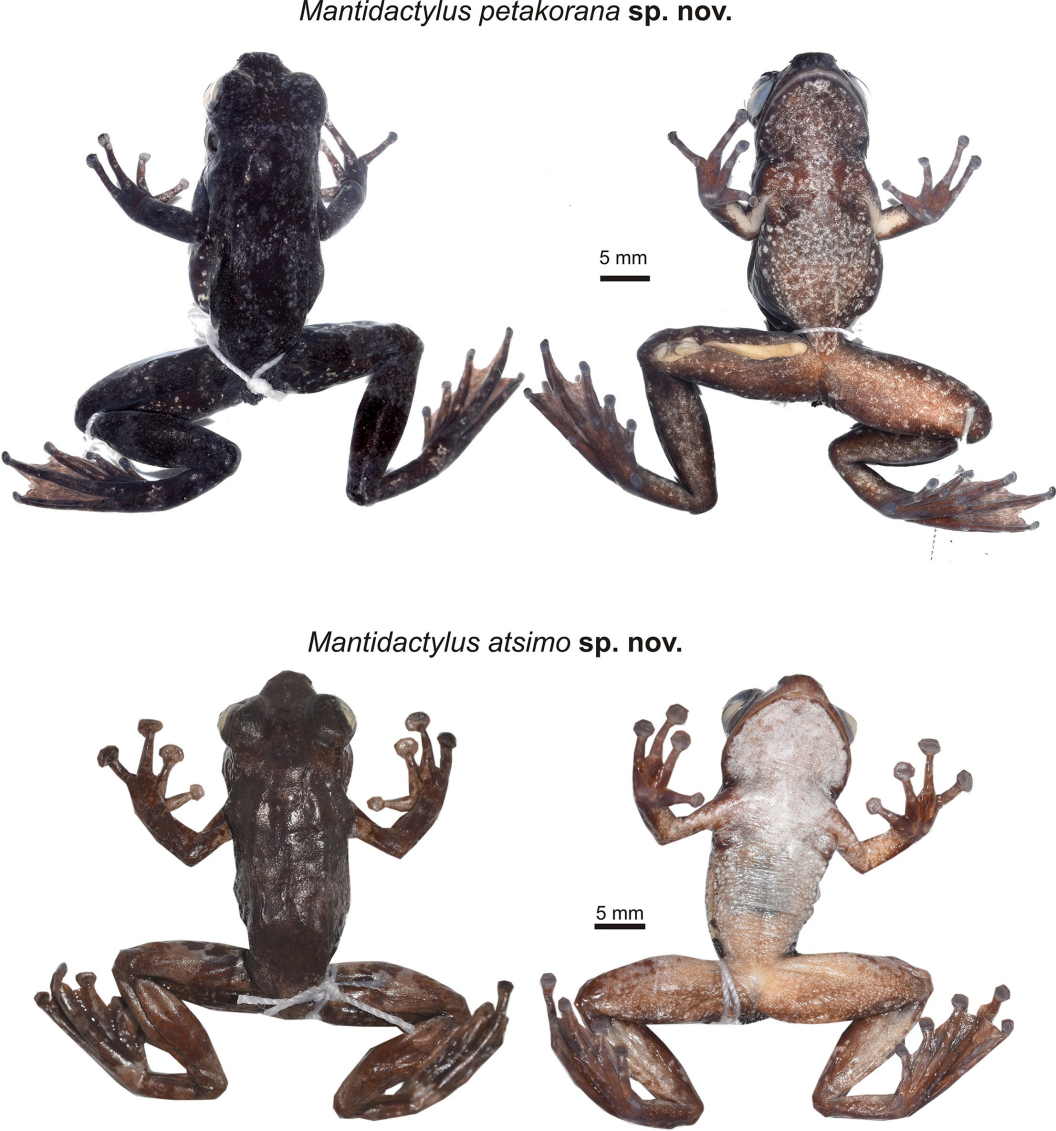
Preserved holotypes of *Mantidactylus petakorona* sp. nov. from Marojejy (ZSM 504/2016), and of *Mantidactylus atsimo* sp. nov. from Andohahela (ZSM 69/2004).

**Fig 8:**
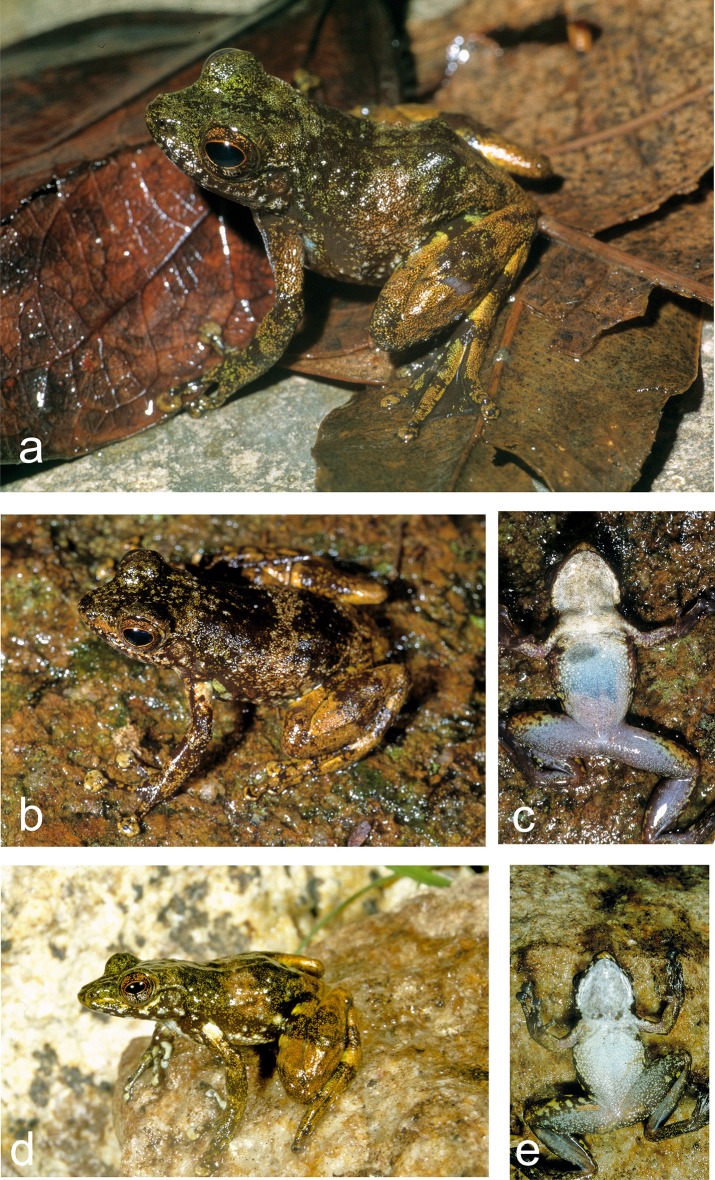
Specimens of *Mantidactylus atsimo* sp. nov. from southeastern Madagascar in life. (a) Specimen from Nahampoana photographed 2001, (b, c) holotype ZSM 69/2004 from Andohahela Camp 1, photographed in 2004, (d, e) specimen from near Tolagnaro (Pic St. Louis) photographed in 1991.

**Fig 9:**
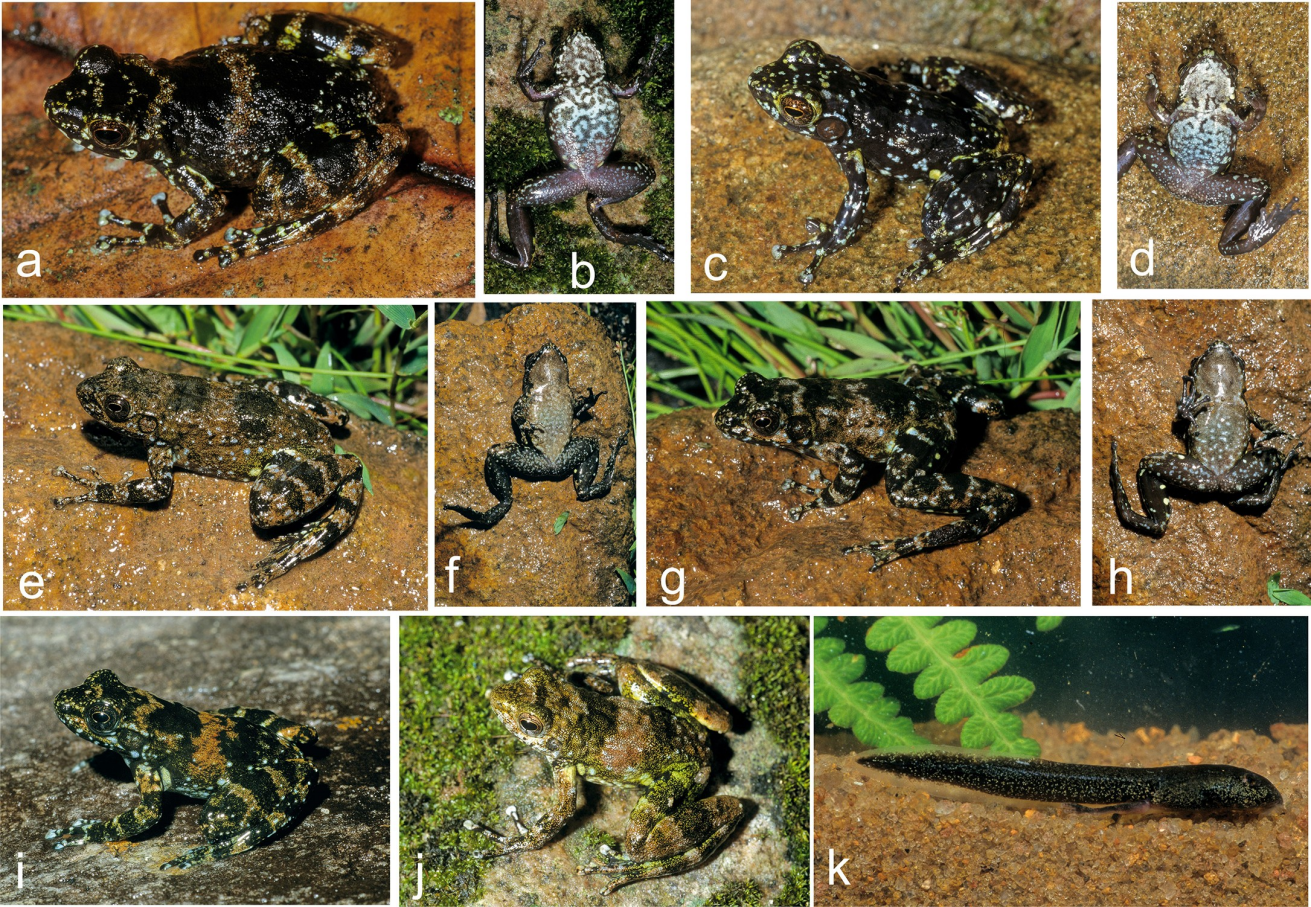
Specimens of *Mantidactylus* sp. Ca48 in life. (a, b) Specimen from Ranomafana, photographed in 2003; (c, d) specimen from Ranomafana, photographed in 2003; (e, f) specimen from Antoetra, photographed in 2003; (g, h) specimen from Antoetra, photographed in 2003; (i) specimen from Itremo, photographed in 2001; (j) specimen from Ranomafana, photographed in 2003; (k) potential tadpole of this species from Antoetra, photographed in 2003.

The first step to achieve an improved taxonomic resolution in *Hylobatrachus* consists of assigning each of the available names, *cowanii* and *lugubris*, to one of the genetic lineages. Preserved syntypes of *M*. *lugubris* are shown in Fig 3 and two living individuals assigned to this species in Fig 4. Living individuals assigned to *M*. *cowanii* are shown in Fig 5. Living and fixed specimens of *M*. sp. Ca52 are shown in Figs 6 and 7, and living and fixed specimens of *M*. sp. Ca49 are shown in Figs 7 and 8, and of *M*. sp. Ca48 in Fig 9.

We here follow the definition of *M*. *lugubris* and *M*. *cowanii* given by Glaw and Vences [15], but we are aware that it might partly be in need of revision. These authors, building on Glaw and Vences [9], defined *M*. *lugubris* (a species without clearly defined type locality) as the main lineage of olive-green coloured stream frogs occurring in the Mantadia/Andasibe region in the Northern Central East of Madagascar, considering that numerous species described by early researchers had been collected in this general region (SVL of syntypes 32–38 mm, see measurements in Table 1). Furthermore, Glaw and Vences [9, 15] defined *Mantidactylus cowanii* (type localities: Ankafana and East Betsileo) as corresponding to a large-sized species that occurs syntopically with *M*. *lugubris* at Mantadia, Vohidrazana and Vohimana, characterised by rather uniform blackish colour with irregular light spotting, in agreement with the *M*. *cowanii* type specimen, described by Boulenger [37] as being dorsally brown, ‘sometimes minutely punctuated with whitish’, with whitish flanks and lateral hindlimbs, measuring 42 mm in SVL.

This definition is not as clear-cut as first hoped. Our samples closest to the type locality of *M*. *cowanii* originate from Antoetra, and correspond to *M*. sp. Ca48, a candidate species that has previously been referred to as *Mantidactylus* sp. aff. *cowanii* ‘small’. This candidate species is widespread, occurring in Manombo, Antoetra, Itremo, Ranomafana, and Ambohitsara (Fig 2). However, large-sized specimens matching the description of *M*. *cowanii* have also been found at Antoetra (Fig 5), but no tissue samples of these individuals are available for molecular analysis. Therefore, we hypothesise that both *M*. sp. Ca48 and the larger-sized *M*. *cowanii* occur at Antoetra, which would support our definition of the latter taxon (see also Andreone et al. [38] who discovered two sympatric *Hylobatrachus* species at Antoetra, but identified them as *M*. *lugubris* and *M*. *cowanii*).

Despite all of the uncertainty surrounding the identity of *M*. *lugubris*, *M*. *cowanii*, and *M*. sp. Ca48, it seems clear to us that neither of the two available names, *M*. *lugubris* or *M*. *cowanii*, refers to either of the genetically divergent lineages, *M*. sp. Ca52 from Marojejy or *M*. sp. Ca49 from the far south of Madagascar. This is based on the following rationale: (1) *M*. *cowanii* was described from Eastern Betsileo, i.e., from the Southern Central East of Madagascar, and no specimens belonging to either of these genetic lineages are known from this part of Madagascar. Furthermore, the size given in the original description (42 mm) clearly exceeds that of specimens from either Marojejy or the extreme southeast of Madagascar (Table 1). (2) *M*. *lugubris* was described without precise locality information, but of the early-described anurans from Madagascar, none is endemic and restricted to northeastern or extreme southeastern Madagascar. Furthermore, the syntypes of *M*. *lugubris* differ morphologically from at least the Marojejy specimens (especially by a longer snout; Table 1).

Consequently, it seems sufficiently clear that none of the two available names refers to the candidate species from the northeast or southeast of Madagascar (*M*. sp. Ca49 and *M*. sp. Ca52). These two candidate species also show some morphological differentiation from other *Hylobatrachus*: the northeastern *M*. sp. Ca52 often has a conspicuously short snout and large eyes, and most individuals of *M*. sp. Ca49 have a uniformly coloured, silvery white ventral side as well as rather large terminal discs on fingers and toes. This combined with their substantial 16S divergence of >>3%, above the threshold typically defining evolutionarily distinct species of neobatrachian frogs [12, 39], led us to propose their formal taxonomic descriptions in the following.

### Taxonomy

**Fig 10:**
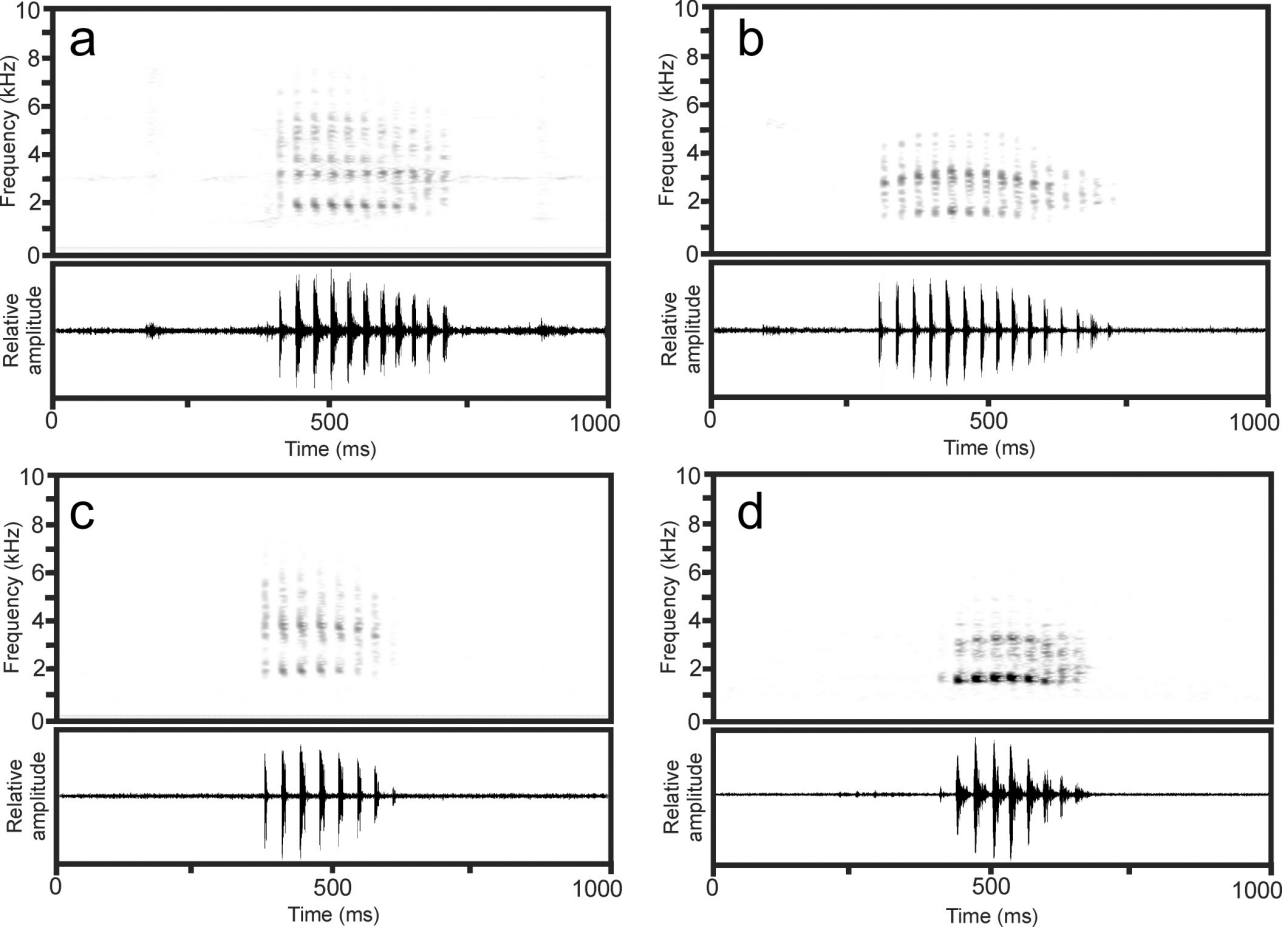
Spectrograms and oscillograms of calls of members of *Mantidactylus (Hylobatrachus)*. (a) A call of *Mantidactylus* sp. (probably *lugubris* but possibly *cowanii)*, recorded on 14 January 1995 near Andasibe at 22.1°C air temperature; (b) a call of *Mantidactylus lugubris*, recorded on 15 January 2016 in Vohidrazana at 17.3°C air temperature; (c) a call tentatively assigned to *Mantidactylus* sp. Ca48, recorded on 10 February 1997 in Ifanadiana at 21°C air temperature; (d) a call tentatively assigned to *Mantidactylus* sp. Ca48, recorded on 29 October 1995 in Ranomafana village at 27.2°C air temperature, bandpass filtered (800–9000 Hz).

**Fig 11:**
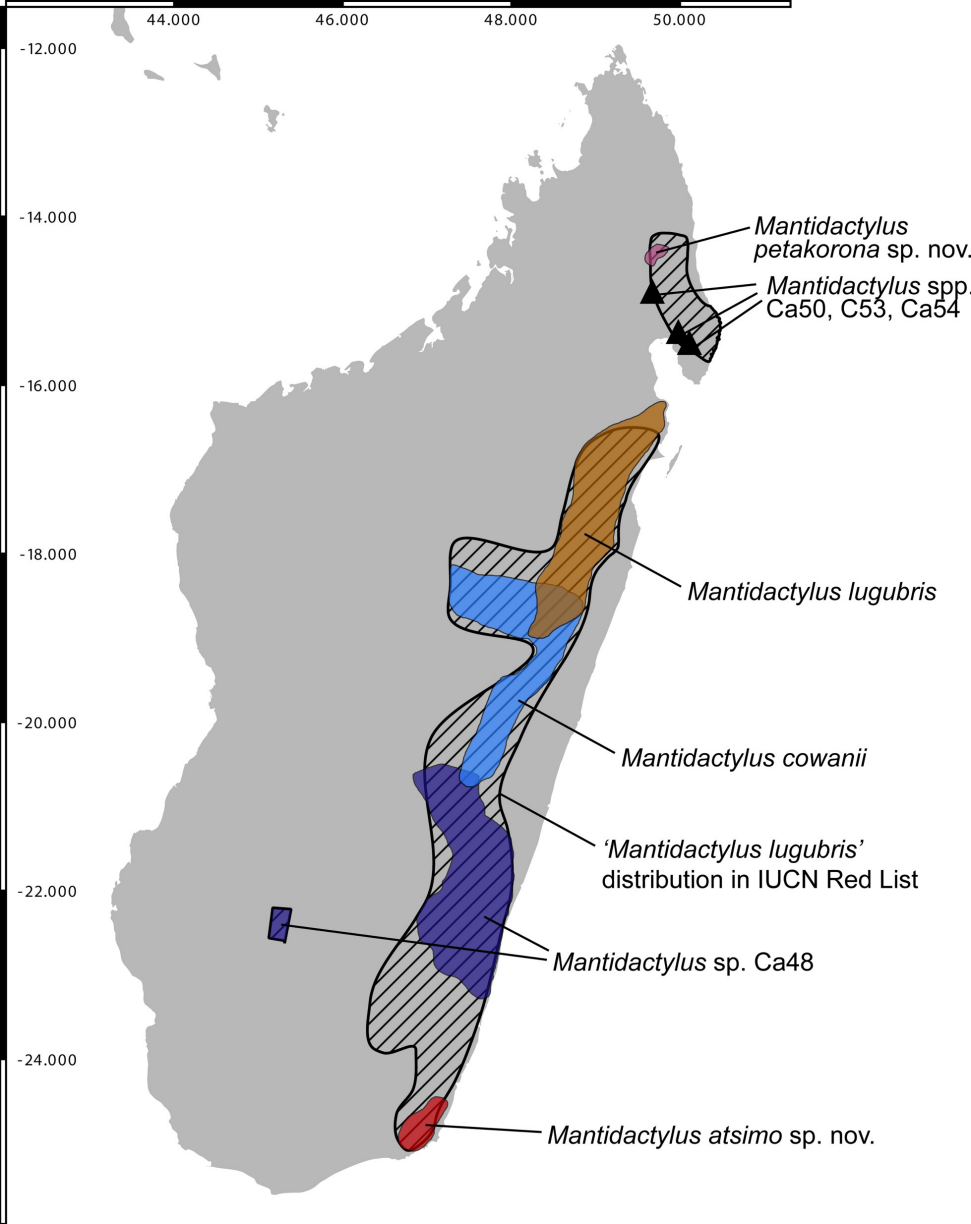
Estimated ranges of *Mantidactylus* species in the subgenus *Hylobatrachus*, compared to the range map of *Mantidactylus (Hylobatrachus) lugubris* on the IUCN Red List (hatched). Note that several *Hylobatrachus* localities of unknown genetic identity were not assigned to any species or candidate species. Ranges of species and candidate species are estimated based on known localities (Fig 2). IUCN Red List map of *M*. *(H*.*) lugubris* from its 2016 re-assessment [23].

***Mantidactylus petakorona* sp. nov**.

urn:lsid:zoobank.org:act:EFD15659-2A5D-4684-8991-EF24ED540208

(Figs 1, 2, 6, 7, 10 and 11, Tables 1 and 2)

#### Remarks

This species was previously considered as *Mantidactylus lugubris* by Glaw and Vences (1994, partim), *Mantidactylus* sp. aff. *lugubris* “Marojejy” by Glaw and Vences (2007: 250–251) and as *Mantidactylus* sp. Ca52 by Vieites et al. (2009, suppl.), Wollenberg et al. (2011, suppl.) and Perl et al. (2014, suppl.).

#### Holotype

ZSM 504/2016 (field number ZCMV 15110), adult female (Figs 6 and 7), collected at Camp 0 in Marojejy National Park (ca. 14.4463°S, 49.7852°E, ~310 m a.s.l.), Sava Region, former Antsiranana Province, northeastern Madagascar, on 15 November 2016, by M. Bletz, M. D. Scherz, J. H. Razafindraibe, A. Rakotoarison, M. Vences, and A. Razafimanantsoa.

#### Paratypes

ZSM 501–503/2016 (field numbers ZCMV 15100, 15104, 15106), ZSM 505/2016 (ZCMV 15111), and UADBA-A uncatalogued (ZCMV 15105, 15121), six specimens with the same collection data as holotype. ZSM 305/2005 (FGZC 2767) collected at Camp Mantella in Marojejy National Park (14.4377°S, 49.7756°E, 481 m a.s.l.) on 14 February 2005 by F. Glaw, M. Vences, and R.D. Randrianiaina; ZFMK 57420, adult (possibly female), collected around a temporary low elevation camp (ca. 300–400 m a.s.l.) in Marojejy National Park on 27 March 1994 by F. Glaw, N. Rabibisoa and O. Ramilison; ZFMK 59909, adult female, collected at a temporary low elevation camp (ca. 300–400 m a.s.l.) in Marojejy National Park on 22–23 February 1995 by F. Glaw and O. Ramilison.

#### Etymology

The specific epithet ‘petakorona’ is a Malagasy word meaning ‘flat nose’, in reference to the distinctly shorter snout of this species. It is treated as an invariable noun in apposition to the genus name.

#### Diagnosis

*Mantidactylus petakorona* sp. nov. differs from all other species of *Mantidactylus*, subgenus *Hylobatrachus*, by a divergence of 5.9–8.7% uncorrected p-distance in a fragment of the 16S gene (uncorrected p-distances of *M*. *petakorona* to *M*. *cowanii* (8.7%), to *M*. *lugubris* (5.6%), to *M*. sp. Ca49, described below (6.5%)). The new species is characterised by the following characters: (1) SVL 27–34 mm, (2) absence of white dorsal and lateral spotting, (3) squared snout in dorsal view, (4) large eyes (ED/HL = 0.32–0.52), (5) almost complete webbing of the fourth toe, and (6) a dark venter.

Among members of the subgenus *Hylobatrachus*, *M*. *(H*.*) petakorona* can be distinguished from *M*. *cowanii* as defined by Glaw and Vences [9, 15] by its distinctly smaller adult SVL (27–34 mm vs. 34–39 mm), shorter relative head length in males (HW/HL 0.86–0.87 vs. 0.90–0.98), generally smaller relative tympanum diameter in females (TD/ED 0.42–0.59 vs. 0.55–0.69, probably due to larger eye size), relatively longer hindlimbs in females (HIL/SVL 1.70–1.87 vs. 1.51–1.63), and lack of rather consistent white dorsal and lateral spotting (vs. presence); from *M*. *lugubris* by a distinctly more squared snout in dorsal view (vs. pointed) and by larger eyes evidenced by smaller TD/ED ratio and larger ED/HL ratio (see Table 1), and relatively longer hindlimbs in females (HIL/SVL 1.70–1.87 vs. 1.52–1.68). For diagnosis against *M*. sp. Ca49, see the description of that species, below.

#### Description of the holotype

Adult female in good state of preservation; SVL 34.0 mm; body relatively slender; head slightly longer than wide, of same width as body; snout rounded, slightly squared in dorsal view, slightly pointed in lateral view; nostrils directed laterally, slightly protuberant, nearer to tip of snout than to eye; canthus rostralis rather indistinct, straight; loreal region slightly concave; tympanum distinct, circular, its horizontal diameter 44% of eye diameter; supratympanic fold slightly distinct; tongue attached anteriorly, distinctly bilobate posteriorly, lobes rounded (right lobe slightly shorter than left lobe); maxillary teeth present; vomerine odontophores distinct, one rounded patch on each side of buccal roof, positioned posteromedial to choana; choanae small, rounded. Arms slender, subarticular tubercles distinct, single; inner metacarpal tubercle and outer metacarpal tubercle not clearly recognisable; fingers without webbing; comparative finger length 1<2<4<3, second finger distinctly shorter than fourth finger; finger discs slightly enlarged. Hindlimbs slender; tibiotarsal articulation reaches slightly beyond the anterior corner of the eye when the hindlimb is adpressed forward along the body; lateral metatarsalia separated by webbing; comparative toe length 1<2<3<5<4; fifth toe only slightly longer than third toe; inner metatarsal tubercle slightly distinct, outer metatarsal tubercle not recognisable; webbing between toes strongly expressed, formula 1 (0), 2i (0.25), 2e (0), 3i (0.5), 3e (0), 4i (0), 4e (0), 5 (0). Dorsal skin smooth; dorsum with slightly distinct dorsolateral folds; ventral skin smooth, including in the cloacal region, where there are no distinct tubercles. For extensive measurements see Table 1.

In preservative (Fig 7), dorsal colour dusky brown from top of head and dorsal abdomen; flanks transitioning from dorsal to ventral from light to dark dirty brown with whitish speckles situated near the hindlimbs; ventral background drab cinnamon with whitish speckles, darker, less speckled colour extending from the attachment of the arm, chin less speckled than the abdomen; dorsal forelimbs a dusky brown, ventral forelimbs centrally translucent surrounded by drab cinnamon, dorsal hindlimbs dusky brown, transition zone from dorsal to ventral surface is speckled with pale buff, ventral hindlimbs drab with pale buff speckles, hindlimbs distinctly less speckled than ventral abdomen; toe tips dusky brown.

In life (Fig 6A–6D), the dorsal background was a blackish granite in colour, with light greenish grey speckling on head and dorsum, discontinuous lateral band pattern on mid dorsum with light greenish grey and light lime green colouring; ventral background a pale grey-brown with extensive white to pale blue mottling; dorsal forelimbs dusky brown-black with light greenish-grey speckling, ventral forelimbs centrally translucent surrounded by brownish olive, dorsal hindlimbs dusky brown-black with distinct light greenish grey bands, ventral hindlimbs brownish olive with white to white-blue flecks. Toe tips lighter in colour. Iris copper coloured.

#### Variation

Morphologically studied paratypes include two males (ZCMV 15106, ZCMV 15100) and two females (ZCMV 15111, ZCMV 15104). There is no clear sexual size dimorphism (males 29.0–31.3 mm, females 27.0–34.0 mm). Femoral glands appear indistinct in male specimens. See Table 1 for detailed morphological measurements. Colour patterns vary between individuals with (1) the extent of lateral banding on the dorsum varying from no apparent bands to multiple distinct bands, (2) lateral bands varying in colour from light greenish to buff yellow, and in the extent of whitish speckling on the ventral abdomen and chin.

#### Distribution and Natural History

Typically found on rocks in small- to medium-sized rainforest streams with moderate flow velocity and on rocks along the stream banks. The call of the species is not known, nor are any data available on its reproductive habits. It is currently only known from Marojejy National Park at low elevation (Fig 2).

***Mantidactylus atsimo* sp. nov**.

urn:lsid:zoobank.org:act:C7B43B19-FB8B-430B-8624-A89536489A09

(Figs 1, 2, 7, 8, 10 and 11, Tables 1 and 2)

#### Remarks

This species was previously considered as *Mantidactylus lugubris* by Glaw and Vences (1994, partim), *Mantidactylus* sp. aff. *lugubris* “Tolagnaro” by Glaw and Vences (2007: 250–251) and as *Mantidactylus* sp. Ca49 by Vieites et al. (2009, suppl.), Wollenberg et al. (2011, suppl.) and Perl et al. (2014, suppl.).

#### Holotype

ZSM 69/2004 (FGZC 116), an ovigerous adult female (Figs 7 and 8), collected between Isaka and Eminiminy (24.7586°S, 46.8542°E, 247 m a.s.l.) in Andohahela, Anosy Region, southeastern Madagascar, between 29 and 31 January 2004 by F. Glaw, M. Puente, M. Thomas, and R. Randrianiaina.

#### Paratypes

ZSM 72/2004 (FGZC 122), an ovigerous adult female, with the same collection data as the holotype. ZSM 149/2004 (FGZC 277) and ZSM 150/2004 (FGZC 281), two ovigerous adult females, collected in Manantantely (24.98°S, 46.92°E, 20–150 m a.s.l.), Anosy Region, southeastern Madagascar, on 8 February 2004 by F. Glaw, M. Puente, M. Thomas, and R. Randrianiaina. ZSM 172/2002 (MV 2001–1476), ZSM 253/2002 (no field number), two ovigerous adult females, and ZSM 173/2002 (MV 2001–1477), a subadult female, collected in Nahampoana (24.9794°S, 46.9839°E, 16 m a.s.l.), Anosy Region, southeastern Madagascar, on 28 December 2001 by M. Vences. ZSM 174/2002 (MV 2001–1483), an ovigerous adult female, collected near the peak of Pic St. Louis (25.0106°S, 46.9731°E, 365 m a.s.l.), Anosy Region, southeastern Madagascar, in December 2001 by M. Vences. ZFMK 52686–52689, a subadult, two females, and a juvenile, respectively, all collected near the peak of Pic St. Louis and the forest near Nahampoana in southeastern Madagascar on 22–27 February 1991 by F. Glaw and M. Vences. ZFMK 53673–53679, seven female specimens, all collected near the peak of Pic St. Louis and the forest near Nahampoana in southeastern Madagascar between 22 December 1991 and 12 March 1992 by F. Glaw and J. Müller.

#### Referred material

ZSM 367–368/2016 (ZCMV 14843–14844), two adult males, collected in Sampanandrano (24.1399°S, 47.0742°E, 539 m a.s.l.), Anosy Region, southern Madagascar, on 16 December 2016 by A. Rakotoarison, E. Rajeriarison, and J. W. Ranaivosolo.

#### Etymology

The specific epithet ‘atsimo’ is a Malagasy word meaning ‘south’ referring to the fact that this is the southernmost species in the subgenus *Hylobatrachus*. It is treated as an invariable noun in apposition to the genus name.

#### Diagnosis

*Mantidactylus atsimo* sp. nov. differs from all other species of *Mantidactylus*, subgenus *Hylobatrachus*, by a divergence of 4.7–6.1% uncorrected p-distance in a fragment of the 16S gene (uncorrected p-distances of *M*. *atsimo* to *M*. *cowani* (6.1%), to *M*. *lugubris* (4.7%), to *M*. *petakorona* (5.5%). The new species is characterised by the possession of the following characters: (1) SVL 25–35 mm, (2) banded dorsal colouration, (3) relatively long snout, pointed in lateral view, (4) moderately sized eyes (ED/HL = 0.35–0.46), (5) fully webbed feet, and (6) whitish venter without dark brown markings. Females also have comparatively shallow snouts.

Among members of the subgenus *Hylobatrachus*, *M*. *(H*.*) atsimo* can be distinguished from *M*. *cowanii* as defined by Glaw and Vences [9, 15] by its generally smaller adult SVL (25–35 mm versus 34–39 mm) and lack of rather consistent white dorsal and lateral spotting (vs. presence); from *M*. *lugubris* by lighter belly colouration, larger brown flecks on males, smaller relative tympanum size in males (TD/ED 0.58–0.66 vs. 0.71–0.91) and females (TD/ED 0.41–0.60 vs. 0.60–0.71), females with a rounded, slightly protruding snout (vs. acute snout); and from *M*. *petakorona* by slightly longer relative snout length in males (END/SVL 0.11–0.12 vs. 0.09–0.10) and typically whitish ventral colouration (vs. dark coloured), discs of third finger broader (pad of third toe ca. twice as broad as finger vs. ca. 1.5 times as broad), snout pointed in ventral view (vs. truncate) in females.

#### Description of the holotype

Adult female in good state of preservation; SVL 34.5 mm; body relatively slender; head slightly longer than wide (HW/HL 0.92), slightly wider than the body; snout rounded in dorsal view, slightly pointed in lateral view; nostrils directed laterally, protuberant, nearer to tip of snout than to eye; canthus rostralis distinct, slightly curved; loreal region concave; tympanum distinct, circular, its horizontal diameter 41% of eye diameter; supratympanic fold slightly distinct; tongue taken as tissue sample; maxillary teeth present; vomerine odontophores distinct, one rounded patch on each side of buccal roof, positioned posteromedial to choana; choanae small, rounded. Arms slender, subarticular tubercles indistinct, single; inner metacarpal tubercle and outer metacarpal tubercle not clearly recognisable; fingers without webbing; comparative finger length 1<2<4<3, second finger distinctly shorter than fourth finger; finger discs distinctly enlarged. Hindlimbs slender with a robust thigh; tibiotarsal articulation reaches the eye when the hindlimb is adpressed forward along the body; lateral metatarsalia separated by webbing; comparative toe length 1<2<3<5<4; fifth toe slightly longer than third toe; inner metatarsal tubercle slightly distinct, outer metatarsal tubercle not recognisable; toes completely webbed, formula 1 (0), 2i (0), 2e (0), 3i (0), 3e (0), 4i (0), 4e (0), 5 (0). Dorsal skin smooth; dorsum without dorsolateral folds; ventral skin smooth on the chin but granular over the abdomen and in the cloacal region; no distinct tubercles in the cloacal region. For measurements see Table 1.

In preservative (Fig 7), dorsal colour chocolate brown, lighter over the head and one band on the mid-body; flanks transitioning from dorsal to ventral from chocolate brown to burnt umber, with a moderately distinct colour border at the junction of the ventral colouration, which is a pale cream over the anterior body and more yellowish posteriorly and on the ventral legs, where it mixes with brown. The forelimbs are dorsally as the trunk in colour, and ventrally cream except on the hands, which are brown. The toe pads are a brown-grey, both on hands and feet. The hindlimbs are banded dark brown, milky brown, and red-brown. When the leg is bent (at rest), these crossbands line up to form consistent bands over thigh, shank, and foot. The hidden surfaces of the legs are chocolate brown as the dorsum, and the anterior thigh also has large blotches of burnt umber bordered in pale cream. The webbing is drab brown in colour.

Colouration in life (Fig 8B and 8C) was much more vibrant and contrasting in colour than in preservative, but the pattern was the same. The dorsal trunk was dark burnt umber with a chocolate-brown band at mid-body and speckled chocolate on the head. A honey-brown stripe was present in the loreal region. The forelimb was as the dorsum in colouration, with a cream spot near the axilla, and a yellow-green marking on the flank beside the axilla. The dorsal hindlimbs were honey-brown cross-banded with burnt umber. The venter was taupe over the posterior abdomen and hindlimbs, bluish over the anterior abdomen, and dirty white on the chin and pectoral region. The iris was bronze.

#### Variation

Individuals morphologically studied in detail include six female paratypes and two males tentatively attributed to *M*. *atsimo* (Table 1). Males appear to be slightly smaller than females (25 and 28 mm vs. 31–35 mm). Femoral glands are moderately distinct in males (Fig 8E). Colour patterns are relatively similar among all ZSM paratypes, including the presence of crossbands on the body and hindlimbs, and the presence of distinct light spots in the axilla. Ventral colouration is more variable, with most specimens having white chins except ZSM 174/2002, 149/2004, and 150/2004. ZSM 149/2004 has an unusual pathology of the right thigh, with a large subcutaneous growth. Specimens from the Anosy mountains that are tentatively assigned to this species differ in possessing dark spots on their venters, white toe tips, and distinct femoral glands in males (ZSM 367/2016 and 368/2016).

#### Natural History

Typically found on rocks in small to medium sized rainforest streams with moderate flow velocity and on rocks along the stream banks, also in heavily degraded forest near the peak of Pic St. Louis. During the day, females were sitting on rocks close to the water level. When disturbed, the frogs jump across the surface of the water at great velocity, coming to rest only at the next available rock (again at the water level). In this manner they were able to cross a stream of several metres width within a few seconds. They avoid diving in the water, probably due to high predation pressure (e.g. by large aquatic crustaceans). Almost all collected specimens were females, suggesting different habits of males and females. The calls and the clutches of the species are unknown. The blackish and elongated tadpoles were roughly described by Glaw and Vences (1994, page 167 and Figs 192, 193, Tad 28) based on individuals from near the peak of Pic St. Louis and Nahampoana. They are exotrophic and live on the ground of the streams. The highly specialised mouthparts without horny beak and labial teeth appear to be a filter apparatus. Metamorphosis was observed in December/January and juveniles measured 10–11 mm SVL.

#### Distribution

Currently known from Andohahela, Manantantely, Nahampoana, and Pic St. Louis, all in southeastern Madagascar. Specimens from Sampanandrano in the Anosy mountains referred to this species require taxonomic clarification, but these expand the distribution of this species considerably northwards.

### Vocalizations in *Hylobatrachus*

Despite being relatively common along rocky streams in Madagascar's rainforests, *Hylobatrachus* are bioacoustically remarkably inconspicuous. Only on few occasions have advertisement calls been recorded. These calls are described in the following, to provide a baseline for future bioacoustic comparisons in this group of frogs.

*Mantidactylus* sp. (probably *lugubris*, but might refer to *cowanii* which also occurs in nearby areas).–Two calls recorded from a male (not collected), sitting on a tree trunk ca. 2 m above the water level of a quietly running stream, on 14 January 1995 near Andasibe (at the border of Analamazaotra reserve) by F. Glaw, at 22.1°C air temperature (one call provided by Vences et al. [40]: CD2, Track 88, Cut 4) each consist of a single short, strongly and regularly pulsed note (Fig 10A) with the following parameters: note duration (= call duration) 314–320 ms; 11 pulses/note; pulse duration varies from 14–18 ms; inter-pulse intervals 12–18 ms; pulse repetition rate is 34–35 pulses/s; dominant frequency 1520–2020 Hz; prevalent bandwidth 1500–7200 Hz. Moderate amplitude modulation is recognisable among pulses, with the initial pulse emitted with much lower energy, followed by 3–4 pulses with high amplitude that decreases slightly in subsequent pulses towards the end of the note. Call repetition rate of reasonably motivated calls unknown.

*Mantidactylus lugubris*.–Two calls recorded at night on 15 January 2016 at Vohidrazana by C. Hutter, at 17.3°C air temperature, from a male confirmed by its typical colour pattern to be *M*. *lugubris* (voucher specimen CRH1293) each consist of a single short, strongly and regularly pulsed note (Fig 10B) and have the following parameters: note duration (= call duration) 428–430 ms; 15 pulses/note; pulse duration varies from 9–14 ms, with initial pulses of a note being the shortest; inter-pulse intervals 18–24 ms; pulse repetition rate ca. 32–35 pulses/s; dominant frequency 1540–1690 Hz; prevalent bandwidth 1400–6500 Hz. Amplitude modulation is recognisable among pulses, with highest energy present in the fourth pulse of the note. Frequency modulation is apparent within notes, with dominant frequency slightly increasing from the beginning to the middle of the note, and continuing with dropping dominant frequency towards the end of the note, reaching a slightly lower level than that of the beginning. Call repetition rate of reasonably motivated calls is unknown.

*Mantidactylus* sp. cf. Ca48.–Calls are tentatively assigned to this candidate species based on the recording localities as in this region of Madagascar (Ranomafana region) only this lineage of *Hylobatrachus* has so far been identified (no calling voucher specimens available).

Seven calls recorded on 10 February 1997 in Ifanadiana by F. Andreone (partly provided in Vences et al. [40]: CD2, Track 88, Cuts 1–3) at 21°C air temperature each consist of a single short, strongly and regularly pulsed note (Fig 10C) and have the following parameters: note duration (= call duration) 217–248 ms (240 ± 11 ms, n = 7), 7–8 pulses/note (n = 7); pulse duration varies from 4–15 ms (10 ± 3 ms, n = 55), with the initial pulse of a note being the shortest; inter-pulse intervals 19–28 ms (24 ± 2 ms, n = 48); pulse repetition rate 32.3–33.8/s (32.7 ± 0.5/s, n = 7); dominant frequency 1860–2050 Hz; prevalent bandwidth 1600–7000 Hz. Slight amplitude modulation is recognisable among pulses, with highest energy present in third and fourth pulses of the note.

Three calls recorded on 29 October 1995 at Ranomafana village by J. Köhler at 27.2°C air temperature (23°C water temperature) each consist of a single short, strongly and regularly pulsed note (Fig 10D) and have the following parameters: note duration (= call duration) 235–284 ms; 8–9 pulses/note; pulse duration varies from 10–14 ms, with initial pulses of a note being the shortest; inter-pulse intervals 19–22 ms; pulse repetition rate app. 29–32 pulses/s; dominant frequency 1540–1690 Hz; prevalent bandwidth 1350–6500 Hz. Amplitude modulation is recognisable among pulses, with highest energy present in third and fourth pulses of the note. Compared to the call from Ifanadiana, pulses appear to be less well spaced, but this is probably due to echo effects in the stony river bed of the Ranomafana river where the recording was obtained. Call repetition rate of reasonably motivated calls is unknown.

## Discussion

### First steps toward resolving the taxonomy of *Hylobatrachus*, and its integrative future

The frogs of the subgenus *Hylobatrachus* are among the most enigmatic members of the genus *Mantidactylus*. Their reproductive habits are poorly known, as are their often poorly recognizable femoral glands, their highly specialised tadpoles [16], vocalization and diet. Only one species was recognised as valid until Glaw and Vences [9] resurrected *M*. *cowanii* from synonymy with *M*. *lugubris*. Genetic evidence revealed that this was still a considerable underrepresentation of the species diversity of this subgenus, and that several new candidate species exist within it [12]. Small sample sizes and the aforementioned poor knowledge concerning these frogs hampered that revision, and only now has it been possible to assemble the modest sample size we report from just the two candidate species addressed here.

In this revision, we have described the two new species, *M*. *petakorona* and *M*. *atsimo*. Both have been recognised as potentially distinct since at least 2007 [15]. *Mantidactylus petakorona*, characterised by a distinct short snout and large eyes, is restricted to Marojejy, and is the northern-most representative of the subgenus, while *M*. *atsimo*, characterised by a typically white belly and dorsal crossbands, as well as complete webbing of its feet, is the southern-most representative. We have here refrained from revisiting the taxonomy of *M*. *cowanii* and *M*. *lugubris*, for which large sample sizes are available. However, we note that the description of the lineage called *M*. sp. Ca48 will require the careful reassessment of the assignment of these names, given its wide distribution and similarity to those species. The identity of the candidate species *M*. sp. Ca50, *M*. sp. Ca53, and *M*. sp. Ca54 will also require future efforts, as those lineages are currently known from only few samples.

Recordings of calls of these frogs are rare because the males are difficult to record. The sparse bioacoustic data available do not contribute much to the understanding of species limits in *Hylobatrachus*, as they provide an inconclusive picture. Comparison of calls referred reliably or tentatively assigned to *M*. *lugubris* with those corresponding to *M*. sp. Ca48 reveals slight differences in numerical parameters (e.g., note duration, number of pulses per note). The greatest difference is that *M*. *lugubris* calls have a larger number of pulses compared to *M*. sp. Ca48 calls (11–15 vs. 7–9 pulses), but the general call structure of all the calls recorded is very similar and usually would not qualify as species-specific differences, particularly not among allopatric populations (see [24]). However, this picture may change once more call recordings become available, and deserves future attention.

While we have succeeded in identifying morphological characters differentiating these species, we have also shown that haplotype sharing in at least some nuclear genes is rather high within this subgenus. It is in this light that we emphasise that future testing may falsify some of our results. Nevertheless, we consider the two new species proposed here likely to be robust, given their high mitochondrial divergence and concordance with morphological differences.

Biogeographically, the genus *Hylobatrachus* presents an interesting pattern that is worthy of cursory remark: The known diversity of this subgenus is distributed from the far southeast of Madagascar to Marojejy in the north, including localities in the highlands of central Madagascar, and an isolated population in western Madagascar (Isalo). It is curious that no representatives have yet been found in any part of the northwestern end of the eastern escarpment, that is, the chain of mountains that runs from Anjanaharibe-Sud northwest to Tsaratanana, then southwest to Manongarivo, and northeast to Sorata. All other subgenera of *Mantidactylus*, except the monotypic *Maitsomantis*, are represented in this region by at least one species, yet for some reason *Hylobatrachus* is apparently not. As fieldwork in this area has been less intense than in eastern Madagascar, there is a chance that *Hylobatrachus* have simply been overlooked. However, it is also evident that *Hylobatrachus* is absent in the well-studied northernmost Malagasy rainforest of Montagne d'Ambre.

### The need for changes to the way the IUCN Red List treats species complexes

Our new data takes us a step closer to resolving the taxonomy of the subgenus *Hylobatrachus*. This is just one of dozens of species complexes that are known among the frogs of Madagascar; recognition of species complexes is subjective, so a precise number for any given system cannot be calculated. At a rough estimate, we suppose that between a third and a fifth of Madagascar’s ca. 360 recognised frog species belong to species complexes, some involving mostly undescribed species (e.g. *Boophis marojezensis* is one of around eight similar-looking frogs [41]), others involving numerous available names and described taxa (e.g. in the genus *Plethodontohyla* [12, 42]). The amphibians of Madagascar are exceptionally well characterised in this regard; in other biodiversity hotspots where DNA barcoding has not yet been broadly applied, such as New Guinea and Borneo, we can expect that far more species complexes are likely to be discovered once the fauna has been genetically characterised.

Given how pervasive species complexes are, and how challenging and time consuming it can be to resolve them, species-directed conservation strategies need to find appropriate ways to assess them in a way that reflects the nature of their taxonomic uncertainty. The current recommendations of the IUCN Red List, as outlined in the Introduction, are (1) to treat species complexes as a single good species and assess it as such, as long as sufficient data are available to do so, or (2) to list the species as DD if there are insufficient data to do so, or (3) to omit the complex from the list altogether [5]. More often than not, this results in the first option, and as the species complex as a whole is invariably more widespread than any single member within it, and often spread over a very large area, complexes tend to be listed as Least Concern [6]. This is an inaccurate reflection of the taxonomic uncertainty of the complex, and neglects the risk that any one lineage within the complex may be facing.

This is what happened with *Mantidactylus (Hylobatrachus) lugubris*. Although data were already published showing that it was a species complex and that the assignment of the name was tenuous [9, 12], the guidelines were followed in our assessment of the species, and it was assessed including locations that were known to refer to candidate species. The definition of the species had already been restricted to one genetic lineage by Glaw and Vences [9], and although that assignment remains uncertain, a Red List assessment based on that more strict definition would have been representative of a single lineage, and therefore have better reflected the real threat status of that species. Alternatively, an assessment as DD would have better represented the taxonomic uncertainty that precluded accurate threat assessment. As it was, the assessment referred to multiple species, including both of the new species we have described here.

As mentioned above, complexes can vary in the amount of available data, from total uncertainty around names to well characterised complexes simply awaiting taxonomic treatment. We argue that the way that species complexes are treated should depend on the degree of complexity and available data:

In rare cases where the complex has been characterised genetically (e.g. through DNA barcoding), candidate species well-established, and the definition of available names restricted as far as possible, we recommend that threat assessment be restricted to omit undescribed members of the species complex, i.e. to refer to a single species, even if there is a small risk that the assignment is inaccurate. Reassignment of one assessment from one name to another is a minor issue, compared to producing wholly non-informative inflated assessments that must be overhauled as soon as any taxonomic progress is made. We note also that the IUCN Red List does allow for the assessment of undescribed species, but these are currently so restrictive (a manuscript describing the species must at least be in preparation [5]) as to only seldom be of any use in dealing with species complexes.

As stated, the above case is rare. Far more frequent is the case where a complex has been recognised, but assignment and restriction of names has not been or cannot yet be attempted. Where these things have not been achieved (e.g. where multiple lineages are known, but it is not clear to which lineage an available name should be applied), data are not adequate for an accurate assessment, and the category DD is appropriate. Yet, a combination of factors, including the policy of treating the whole complex as a single good species, and policies that strongly discourage the use of DD, mean that this category is almost never used in such cases. We argue that taxonomic uncertainty alone should be ample justification for the use of the DD category. Even with superb data on the distribution of the complex as a whole, with which it could be assessed as a single species (and would be assessed, following to the current guidelines), such an assessment fails to capture the threats facing any one of the constituent species-level lineages, named or unnamed. DD, on the other hand, highlights the fact that, for conservation of any member of the complex to be successful, taxonomic research is first needed. This would place appropriate weight on the importance of taxonomic accuracy in Red List assessments.

Following this proposal would produce more, and not fewer, DD species in the IUCN Red List. For example, numerous Madagascan frogs currently listed as LC are known to constitute species complexes, such as *Mantidactylus (Brygoomantis) betsileanus* [43], *M*. *(B*.*) curtus* [44], *Guibemantis (Pandanusicola) pulcher* [12, 45, 46], *Rhombophryne laevipes* [47], and *Scaphiophryne calcarata* [48]—we argue that these species, and many more, should be assessed as DD. We anticipate that this proposal will not be met with unanimous approval, because DD species tend to be omitted from conservation prioritisation, and amphibians have a particularly large proportion of DD species already [49, 50]. Concern over this fact has led to a concerted effort to estimate the threat status of amphibians by extrapolating the available data from DD species (e.g. [49, 51–54]). Extrapolation of data that are definitionally deficient is risky, and the accuracy of such approaches may be questionable, although this problem appears to have been best addressed by the most recent study of this kind [49]. However, with few exceptions (e.g. [54]) these studies fail to place emphasis on the fact that the best solution to the deficit in the data of these species is prioritising the basic research needed to bring them out of the DD category. Often, this will be taxonomic research, including renewed field exploration and collection, which, we argue, is as it should be, because taxonomy truly is the foundation of the IUCN Red List—and indeed much species-directed conservation planning—and assessments can only be as reliable as their underlying taxonomy.

In fact, a further argument against the current practice is that it gives policy-makers a false sense of a scientifically validated species list in which each species’ threat has been reliably clarified. This immediately translates into a reluctance to support field exploration and collection activities for taxonomic purposes, be it by not allocating funds to these research activities, or—more commonly—by refusing the necessary permits. Red Lists that do not assign DD categories where it would be necessary, and thus do not acknowledge the need for taxonomic exploration, thus contribute to the perpetuation of the very taxonomic impediment that hampers efficient threat assessment.

If we wish to reduce the fraction of DD species, we must pour more resources into collecting the required data on them, rather than simply discouraging the use of DD and extrapolating from inadequate data to place the species into other categories. Taxonomic uncertainty makes an assessment fundamentally inaccurate, and DD is the most appropriate available category to represent that uncertainty.

### Revising and refining the IUCN Red List assessments of *Hylobatrachus* species, and the future for yet undescribed candidate species

Following the description of the two new species provided here, we can also suggest modifications of the IUCN Red List assessment of all four species. *Mantidactylus (Hylobatrachus) lugubris*, as currently understood, is distributed from Ambatoroma and Ambodirafia in the north Central East, to Vohimana, Vohidrazana, and Mantadia in the Central East; all other regions currently included in the IUCN status of that species refer to other (candidate) species (Fig 11). As such, it has an Extent of Occurrence (EOO) of ca. 7000 km^2^, although it probably occurs more widely in, for example, the poorly surveyed Zahamena National Park. As is currently included in its assessment, the species ‘requires clear streams and so cannot survive in fully transformed agricultural landscapes,’ and its habitat is experiencing on-going habitat decline. It therefore qualifies for a status of Vulnerable under IUCN Red List criterion B1ab(iii). This suggested re-assessment constitutes a major restriction of the species, omitting the unnamed candidate species.

*Mantidactylus petakorona* is found in Marojejy National Park and is currently not known from any other locations. It occurs in streams at low-elevation. We here follow the assessments for other species from this area, e.g. *Rhombophryne savaka*, in considering the species Endangered under criterion B1ab(iii), due to an estimated EOO of < 1000 km^2^, records from a single threat-defined location, and on-going decline in the extent and quality of appropriate habitat. The species should be searched for in nearby areas, such as Anjanaharibe-Sud Special Reserve and Ambolokopatrika to establish its range.

*Mantidactylus atsimo* is found in five threat-defined locations in southeastern Madagascar, including Anosy, Andohahela, Manantantely, Pic St. Louis, and Nahampoana. These span an estimated EOO of ca. 4000 km^2^. Throughout this area, there is, however, dramatic habitat decline, with extensive deforestation. This species therefore currently qualifies as Endangered under criterion B1ab(iii). An additional location would perhaps move it toward Vulnerable, but at present we prefer to err toward Endangered due to the extent of deforestation in this area. We recommend also that further surveys search for this species or other members of the complex to the north of its known range.

The remainder of the recognised candidate species within the subgenus *Hylobatrachus* cannot be assessed while they remain undescribed. This too emphasises the importance of taxonomic research, as well as the importance of continued field collections, in enabling conservation. Species that are undescribed cannot be adequately protected. Recognition of candidate species does not constitute description, and while candidate species can be included on faunistic lists to lend weight to the importance of protecting certain areas (e.g. [33, 55, 56]), they remain preliminary and unavailable for species-level management; although the IUCN does have provisions to assess these species, as we have outlined above, these are untenable for the majority of cases. The extensive availability of characterised candidate species of reptiles and amphibians is exceptional in Madagascar [12, 14, 57], giving conservation on the island an edge, but the importance of taxonomic assessment of these species remains unabated. Elsewhere, DNA barcoding can also be used as a first line for species discovery, but except at landscape conservation levels, taxonomic description of the discovered species will be needed to ensure their protection.
